# Supplementation with *Lactiplantibacillus plantarum* CNPC003 and *Pilosocereus gounellei* Flour Enhances the Properties of Goat Cream Cheese

**DOI:** 10.3390/microorganisms13020254

**Published:** 2025-01-24

**Authors:** Daniela Karla Medeiros Vasconcelos, Evandro Leite de Souza, Márcia Gabrielle Silva Viana, Maria Isabel Ferreira Campos, Lorena Lucena de Medeiros, Lary Souza Olegário, Mércia de Sousa Galvão, Karina Maria Olbrich dos Santos, Antônio Silvio do Egito, Marta Suely Madruga, Marcos dos Santos Lima, Tatiane Santi Gadelha, Maria Teresa Bertoldo Pacheco, Kataryne Árabe Rimá de Oliveira, Maria Elieidy Gomes de Oliveira

**Affiliations:** 1 Post-Graduate Program in Nutrition Sciences, Department of Nutrition, Center of Health Sciences, Federal University of Paraíba, João Pessoa 58051-900, PB, Brazil; dani-karla@hotmail.com (D.K.M.V.); mgabisviana@gmail.com (M.G.S.V.); 2 Laboratory of Food Microbiology and Biochemistry, Department of Nutrition, Center of Health Sciences, Federal University of Paraíba, João Pessoa 58051-900, PB, Brazil; kataryne.arabe@academico.ufpb.br; 3 Laboratory of Protein and Peptide Chemistry, Department of Molecular Biology, Center for Exact Sciences and Nature, Federal University of Paraíba, João Pessoa 58051-900, PB, Brazil; belcampos.va@gmail.com (M.I.F.C.); santi.tatiane@gmail.com (T.S.G.); 4 Flavor Laboratory, Technology Center, Federal University of Paraiba, João Pessoa 58051-900, PB, Brazil; lorenalucena@live.com (L.L.d.M.); laryolegario@hotmail.com (L.S.O.); merciagalvao@gmail.com (M.d.S.G.); msmadruga@uol.com.br (M.S.M.); 5 Brazilian Agricultural Research Corporation (EMBRAPA), Rio de Janeiro 23020-470, RJ, Brazil; karina.dos-santos@embrapa.br; 6 Embrapa Goats and Sheep, Northeast Regional Center, Campina Grande 58428-095, PB, Brazil; antoniosilvio.egito@embrapa.br; 7 Department of Food Technology, Federal Institute of Sertão Pernambucano, Petrolina 56302-100, PE, Brazil; marcos.santos@ifsertao-pe.edu.br; 8 Center for Food Science and Quality, Institute of Food Technology (ITAL), Campinas 13073-001, SP, Brazil; mtb@ital.sp.gov.br; 9 Laboratory of Bromatology, Department of Nutrition, Center of Health Sciences, Federal University of Paraíba, João Pessoa 58051-900, PB, Brazil; mego@academico.ufpb.br

**Keywords:** goat cheese, probiotic, Cactaceae, fermentation, technological profile, physicochemical profile

## Abstract

This study evaluated the impacts of *Lactiplantibacillus plantarum* CNPC003 and xique-xique flour supplementation on the technological, physicochemical, nutritional, and sensory properties of goat cream cheese over 21 days of refrigerated storage. Four cheese formulations were prepared: a control (CC), one with *L. plantarum* CNPC003 (PC), one with xique-xique flour (XC), and one with *L. plantarum* CNPC003 and xique-xique flour (PXC). XC and PXC had a yellowish-green hue with less brightness. PC and PXC were less firm and adhesive with greater elasticity, cohesiveness, and gumminess, and they had reduced total protein and increased total free amino acids (*p* < 0.05) during storage. The contents of specific volatile compounds increased in PXC during storage. PXC had higher *L. plantarum* counts than PC on day 21 of storage. PC and PXC had distinct colors and textures and were well accepted regarding sensory attributes. Xique-xique flour and *L. plantarum* CNPC003 supplementation positively impact the nutritional and functional characteristics of goat cream cheese without negatively affecting the technological and sensory attributes.

## 1. Introduction

Goat milk has a rich nutritional composition that has many benefits for human health [1]. Interest in developing new products using goat milk has been linked to its high nutritional value and to the aim of improving the sensory acceptance of goat dairy products through technological processing [2,3].

Probiotics are commonly used in dairy products due to the capability of these matrices to ensure probiotic survival during storage without negatively affecting the quality of these products [4]. Including lactic acid bacteria strains with probiotic aptitudes in dairy products, especially those belonging to the *Lactobacillus* genus, could improve technological and sensory aspects in addition to providing benefits to consumer health [5]. Supplementation with probiotic cultures may also contribute to aroma development and minimize the unpleasant aroma of goat products typically perceived by some consumers [6].

*Lactiplantibacillus plantarum* cultures have been commonly associated with health claims [7], such as improving the balance of intestinal microbiota [8] and the immune system [9]. The strain *L. plantarum* CNPC003 isolated from goat milk has shown aptitude for use as a probiotic candidate in foods [10]. *L. plantarum* CNPC003 produces exopolysaccharides (EPSs) with antioxidant properties [11], keeps high survival rates during storage, and simulates gastrointestinal digestion in probiotic non-fermented blended beverages without negatively affecting their physicochemical and sensory parameters during storage [12].

In addition to supplementation with probiotic bacteria, using ingredients rich in fiber, phenolic compounds, and minerals, such as cacti recognized as unconventional food plants, could also be a potential technological strategy to add nutritional and bioactive activities to goat dairy products. One potential ingredient to incorporate into goat dairy products could be *Pilosocereus gounellei* A. Weber ex K. Schum. Bly. Ex *Rowl*, a cactus popularly known as xique-xique and widely adapted to the dry climate of the Brazilian Caatinga biome [13]. In vivo studies have demonstrated the gastroprotective [14] and anti-inflammatory effects of xique-xique [15]. A few studies have investigated the use of xique-xique in food products, such as juice [16], goat yogurt [17], and cookies [18]. These studies highlight the versatility and potential of xique-xique as an innovative ingredient in the development of food products, particularly due to its unique nutritional profile and bioactive properties. By incorporating xique-xique into various formulations, it is possible to create functional foods that not only meet consumer demands for healthier options but also add value to this underutilized resource, promoting sustainability and regional biodiversity.

The functional properties of goat cheese, especially as a food matrix and source of varied probiotic bacteria, have been reported [19,20]. However, research regarding supplementation with the potentially probiotic *L. plantarum* CNPC003 strain in dairy products has been scarce. This study explores the potential of using *L. plantarum* CNPC003 and xique-xique flour in goat cream cheese, evaluating their impacts on technological, physicochemical, nutritional, and sensory properties during refrigerated storage.

## 2. Materials and Methods

### 2.1. Raw Material and Ingredients

Cladodes of xique-xique (*P. gounellei* A. Weber ex K. Schum. Bly. Ex *Rowl*) were obtained from a private cultivation area in Boa Vista, PB, Brazil. Prof. Dr. Leonardo Person Felix conducted plant identification, and a certified species sample was deposited in the Prof. Dr. Jaime Coelho Morais Herbarium at the Federal University of Paraíba. The collection process was authorized by the Brazilian Biodiversity Information System (n. 62681) and the National Genetic Heritage Management System (SISGEN, n. AA17429).

Goat milk (pasteurized at 65 °C for 30 min) was purchased from a Goat and Sheep Breeders Association (Zabelê, PB, Brazil). Freeze-dried probiotic culture of *L. plantarum* CNPC003 (formerly named *L. plantarum* B12) (Genetic Heritage: BRMCTAA179), belonging to the “Microorganisms of Interest to the Food Industry Collection” of Embrapa—Goats and Sheep (Sobral, CE, Brazil), was previously isolated from goat milk. This strain was chosen due to its promising characteristics for application in foods as a novel probiotic, being identified using molecular techniques [10]. Rennet liquid coagulant HA-LA (Microbial Chemosin—*Aspergillus niger* var. *awamori*; coagulant power 1:3000/75 IMCU) was purchased commercially from CHR Hansen (Valinhos, SP, Brazil). Xanthan gum was purchased from Leve Crock^®^ (Piraí do Sul, PR, Brazil), and calcium chloride (CaCl_2_) P.A. from FMaia^®^ Ltd. (Cotia, SP, Brazil).

### 2.2. Processing of Xique-Xique Flour

Xique-xique flour was prepared according to Machado et al. [18]. Xique-xique cladodes were washed with running water and sanitized with immersion in chlorinated water (100 ppm/15 min). The husks were removed, and the pulp was separated from the central stalk. The central stalk was cut into 1 cm thick slices and dried in an oven with air circulation (40 ± 1 °C) until reaching 4% moisture. After drying, the dried material was ground with a knife mill (Willey, Solab^®^, Piracicaba, SP, Brazil) and screened in a 100-mesh vibrating sieve. The flour was exposed to ultraviolet (UV) light for 15 min to reduce potential microbial contamination before being vacuum-sealed in sterile polyethylene bags (approximately 100 g per bag), wrapped in aluminum foil, and frozen (−20 ± 1 °C) until cheese production. Before being added to the goat cream cheese formulations, the xique-xique flour underwent microbiological evaluation, which confirmed the absence of microbial contamination and its suitability for use as an ingredient in the processed cheeses.

### 2.3. Inoculum of L. plantarum CNPC003 and Goat Cream Cheese Preparation

The inoculum was prepared by diluting 0.1 g of freeze-dried *L. plantarum* CNPC003 in 10 mL of powdered goat milk (Caprilat^®^, Nova Friburgo, RJ, Brazil) previously reconstituted in sterile water (0.13%, *w*/*v*), followed by incubation for 22 h (stationary phase) at 37 °C, corresponding to a viable cell count of 8–9 log CFU/g when plated on de Man, Rogosa, and Sharpe (MRS) agar (Oxoid, Basigstoke, UK) supplemented with 0.5% cysteine (Sigma-Aldrich, St. Louis, MO, USA).

The cheeses were prepared according to a previously described method [21]. Four different cheese formulations were prepared: CC—control goat cream cheese (without *L. plantarum* CNPC003 or xique-xique flour supplementation); PC—goat cream cheese supplemented with 10 mL of the inoculum of *L. plantarum* CNPC003 per L of milk (0.01%, *v*/*v*); XC—goat cream cheese supplemented with 1 g of xique-xique flour per 100 g of clot (1%, *w*/*w*); and PXC—goat cream cheese supplemented with *L. plantarum* CNPC003 and xique-xique flour in the aforementioned proportions.

Initially, the goat milk, which had been previously pasteurized, was subjected to an additional thermal treatment at 90 °C for 10 min and cooled to 37 °C to allow for the addition of the ingredients described below. The inoculum of *L. plantarum* CNPC003 was added at a concentration of 10 mL/L (PC and PXC) and calcium chloride solution (0.04 mL/L), and liquid rennet (0.1 mL/L) was added as recommended by the manufacturer (Chr. Hansen). The rennet previously dissolved in filtered potable water (1:1) was added to the milk, and a new agitation was performed for the uniform distribution of ingredients.

**Figure 1:**
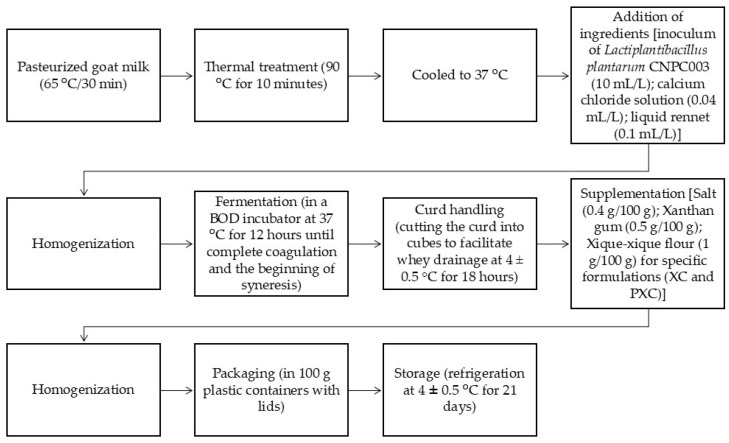
A flowchart of the processes for manufacturing goat cream cheese formulations.

The milk was kept in a BOD incubator (Biochemical Oxygen Demand—Marconi, MA415, Piracicaba, SP, Brazil) at a controlled temperature (37 ± 1 °C) for approximately 12 h for undergoing lactic fermentation. The endpoint of the process was defined by complete milk coagulation and the beginning of syneresis. The formed clot was cut into cubes to allow for draining (4 ± 0.5 °C) for 18 h. Subsequently, the clot was supplemented with salt (0.4 g/100 g) and xanthan gum (0.5 g 100/g). For XC and PXC, 1 g of xique-xique flour per 100 g of clot was added. After homogenization, the clot was packed in plastic containers with lids (100 g) and stored under refrigeration (4 ± 0.5 °C) for 21 days (Figure 1).

The cheeses were processed in three independent experiments and analyzed in triplicate on days 1, 7, 14, and 21 of storage, except for the sensory analyses, which were performed on day 7 of storage, and the determination of fatty acids, sugars, organic acids, and volatile compounds, which were performed on days 1 and 21 of storage.

### 2.4. Technological, Physical, and Physicochemical Parameters of Cheese

The goat cream cheese yields were measured according to a previously described method [22] and expressed as fresh cheese weight (g) per 10 L of milk (used for cheese production). Texture parameters, such as firmness, elasticity, adhesiveness, gumminess, and cohesiveness were evaluated using a double compression test, as described by Barbosa et al. [23]. The analysis was performed with a TA-XT2^®^ texturometer (Stable Micro Systems, Haslemere, UK) equipped with an acrylic cylinder probe (25 mm). The test conditions included a compression of 1 cm and a speed of 1 mm/s. Samples were placed in cylindrical containers (2 cm height and 5 cm diameter) and tested at a controlled temperature of 10 ± 1 °C, and they were removed from refrigeration shortly before the analysis. During the Texture Profile Analysis (TPA) tests, a typical compression of 50% of the sample’s initial height was applied. Data were collected and processed using the Texture Expert software for Windows^®^, version 1.20. Color determination was performed using the CIELab system with labels L* (luminosity), a* [chromaticity (−) green/(+) red], and b* chromaticity [(−) blue/(+) yellow)] in a CR 400^®^ colorimeter (Konica Minolta, Ramsey, NJ, USA). Measurements were performed immediately after cheese was removed from the packaging [24]. The water activity (a_w_) was determined at 25 °C using an Aqualab^®^ device (model CX-2 Water Activity System^®^, Washington, DC, USA). pH, titratable acidity (in lactic acid), moisture, ash, and protein (conversion factor of 6.38) determination was performed using standard procedures [25]. Lipid content determination was performed according to Folch, Lees, and Stanley [26].

### 2.5. Determination of Sugars and Organic Acids in Cheeses

Contents of sugars (glucose, lactose, and galactose) and organic acids (lactic, acetic, and propionic acid) were determined with High-Performance Liquid Chromatograph (HPLC) as previously described [27]. During the analysis, the Agilent Hi-Plex H column (300 mm × 7.7 mm) with a particle size of 8.0 μm and the PL Hi-Plex H guard column (5 mm × 3 mm) (Agilent Technologies ^®^, Santa Clara, CA, USA) were maintained at 50 °C, and the injection volume was 10 μL with a flow rate of 0.5 mL/min, a mobile phase of 4.0 mM H_2_SO_4_ in ultrapure water, and a run time of 20 min. The data obtained were processed using the OpenLAB CDS ChemStation software—Edition TM 2.8 (Agilent Technologies^®^). The glucose and lactose standards were obtained from Sigma-Aldrich, the galactose standard was obtained from Chem Service (West Chester, PA, USA), and organic acid standards were obtained from Vetec Química Fina (Rio de Janeiro, RJ, Brazil), all with a purity of ≥99%. Ultrapure water was obtained using a MilliQ^®^ system (EMD Millipore, Burlington, MA, USA), and sulfuric acid was obtained from Merck (Darmstadt, Germany). Results were expressed in g/100 g of cream cheese.

### 2.6. Protein Characterization of Cheeses

#### 2.6.1. Determination of Soluble Protein

The soluble protein content in cheese samples was determined according to Bradford [28]. After homogenization and rest for 10 min, samples were read at 595 nm in a spectrophotometer (Ultrospec 1100 pro Amersham Biosciences, Amersham, England, UK). A bovine serum albumin (BSA) curve was used as a standard.

#### 2.6.2. Electrophoretic Profile

Electrophoretic profiling was performed according to Laemmli [29]. The application gel (stacking gel) was prepared at a concentration of 7.5 to 17.5% polyacrylamide in 0.5 M Tris—HCl (Tris(hydroxymethyl)-aminomethane) buffer (Sigma-Aldrich), pH 6.8, and 1% SDS (sodium dodecyl sulfate) (Sigma-Aldrich). The separation gel was mounted, forming a gradient of 3.5% polyacrylamide in 3 M Tris-HCl buffer, pH 8.8, and SDS at 1%. The samples were submitted to protein extraction at two storage periods as previously described [30]. Each run was performed under constant amperage (25 mA), and the gel was removed from the plate at the end of the run, fixed in 12.5% TCA (Trichloroacetic acid, Sigma-Aldrich) for 1 h, and stained with Coomassie brilliant blue R-250 to 0.005%. Removal of excess dye was performed with a bleaching solution of methanol, acetic acid, and water (1:3.5:8, *v*/*v*/*v*/*v*). The molecular weights of the cheese protein fractions were compared using a molecular weight marker ranging from 12 to 225 kDa (Amersham Rainbow Marker—GE Healthcare^®^, Amersham, UK, full range).

#### 2.6.3. Total and Free Amino Acid Profiles

The total amino acid profile was determined with a pre-column derivatization of amino acids released after acid hydrolysis (6 mol/L) under heating (110 °C/20 h) according to White et al. [31]. Free amino acids were determined without performing acid hydrolysis [32]. Derivatized amino acid analysis was performed with HPLC. The amino acids were dissolved in diluent and introduced into the RP-HPLC column with a UV detector at 254 nm (Shimadzu Organismoration, Tokyo, Japan) equipped with a C18 Luna/Phenomenex column (250 mm × 4.6 mm × 5 µm; Phenomenex Inc., Torrence, CA, USA). Amino acids were quantified by comparison using Thermo Amino Acid Standard Scientific (Rockford, IL, USA) and DL-2-aminobutyric acid (Sigma-Aldrich) as an internal standard.

### 2.7. Fatty Acid Profile

Fatty acid methylation was performed according to Molkentin and Precht [33]. Initially, 0.5 g of lyophilized sample was subjected to fatty acid methyl ester extraction with 2 mol/L KOH and 1.25 mol/L HCl solutions diluted in methanol. The detection of fatty acid esters was performed in a gas chromatograph (Varian 430-GC, Santa Clara, CA, USA) coupled with a flame ionization detector (FID) and a fused silica capillary column (SPTM—2560, Supelco, Bellefonte, PA, USA) with 100 m × 0.25 mm and 0.20 μm film thickness dimensions. Helium was used as the carrier gas (flow rate of 1 mL/min).

The initial oven temperature was 40 °C for 2 min, increasing by 10 °C/min until reaching 180 °C (remaining for 30 min), followed by another increase at a rate of 10 °C/min until reaching 240 °C (remaining for 10 min). Injector and detector temperatures were maintained at 240 and 250 °C, respectively.

Aliquots of the esterified extract (1.0 μL) were injected into a Split/Splitless injector (Split 1:100). Chromatograms were recorded using Galaxie Chromatography Data System software. Fatty acids were identified by comparing the retention times of the methyl esters of the samples with Supelco ME19—Kit (Boston, MA, USA) standards. The fatty acid results were expressed as g 100/g. From these data, the atherogenicity index (AI), thrombosis index (TI), desirable fatty acids (DFA), and hypercholesterolemic saturated fatty acids (HSFA) were calculated [34] according to Equations (1)–(4).

(1) $$AI=\frac{\left(C12:0+4\;\times \;C14:0+C16:0\right)}{\left[\Sigma MUFA+\Sigma PUFA\left(n-6\right)\;and\;\left(n-3\right)\right]}$$

(2) $$TI=\frac{\left(C14:0+C16:0+C18:0\right)}{\left[0.5\times \Sigma MUFA+0.5\times \Sigma PUFA\left(n-6\right)+3\times \Sigma PUFA\left(n-3\right)+\frac{\left(n-3\right)}{\left(n-6\right)}\right]}$$

DFA = MUFA + PUFA + C18:0(3)

HSFA = C12:0 + C14: 0 + C16:0(4)

### 2.8. Profile of Volatile Compounds

The extraction of volatile compounds was performed using a solid-phase microextraction technique (SPME) with a Supelco SPME device (Bellafonte, PA, USA). The fiber used was a 50/30 μm layer of Divinylbenzene/Carboxene/Polydimethylsiloxane (DVB/CAR/PDMS), activated according to the manufacturer’s instructions.

Cheese samples (20 ± 0.1 g) were placed in 100 mL glass vials and hermetically sealed with screw caps containing a Teflon-coated septum. After equilibration at 45 °C for 20 min, the fiber was exposed to the headspace for 40 min under agitation. The desorption time was 10 min, and separation was performed on a 7890B gas chromatograph coupled to a mass detector (Agilent Technologies 5977B, Little Falls, DE, USA). A VF-5 MS fused silica capillary column was used (30 m × 0.25 mm × 0.25 μm). The oven was initially heated to 40 °C for 10 min, and the temperature was then ramped up to 240 °C at 5 °C/min and maintained for 11 min. The total run time was 61 min. The injector temperature was maintained at 250 °C. A mass spectrometer was used in electronic impact mode with a source temperature of 230 °C, an ionizing voltage of 70 eV, and a scan range of 35 to 350 *m*/*z* with 3.33 scans.

The identification of compounds was based on the analysis of fragmentation patterns displayed in the mass spectra, as confirmed by comparing mass spectra with those present in the database provided with the NIST equipment (National Institute of Standards and Technology, Gaithersburg, MD, USA) and by comparing retention rates with those of known compounds in a homologous standard solution of n-alkanes (C8–C30).

### 2.9. Hygienic–Sanitary Quality Control and Viable Cell Counts of L. plantarum CNPC003

The microbiological parameters of goat cream cheese samples were evaluated using standard procedures [35]. The assessment of the microbiological quality parameters indicative of hygienic–sanitary quality control consisted of counting coagulase-positive staphylococci and *Escherichia coli* and detecting the presence of *Salmonella* ssp. according to the procedure set by the Brazilian legislation [36]. The viable cell counts of *L. plantarum* CNPC003 in the cheeses (XC and PXC) were determined throughout the storage period. Enumeration was performed by plating on de Man, Rogosa, and Sharpe (MRS) agar (HiMedia, Mumbai, India) supplemented with 0.5% cysteine. Plates were incubated anaerobically at 37 ± 1 °C for 48 h using the Anaerogen system (Anaerogen, Oxoid, Basingstoke, Hampshire, England, UK). The viable cell counts were expressed as log CFU/g [35].

### 2.10. Sensory Analysis

This study was previously submitted and approved by a Human Research Ethics Committee (Health Sciences Center, Federal University of Paraíba, PB, Brazil; protocol number CAAE: 79748617.2.0000.5188) recognized by the Brazilian National Research Ethics Commission.

The consumer acceptance test was performed with 102 consumers of goat dairy products (26 men and 77 women, 20–40 years old; average age of 32 years old) who evaluated the appearance, color, texture, aroma, and taste and graded the overall acceptance using a structured nine-point hedonic scale (1 = extremely disliked; 5 = neither liked/disliked; 9 = extremely liked). Intent to purchase was assessed using a 5-point structured hedonic scale (1 = would never buy; 3 = might buy/maybe not buy; 5 = would certainly buy). Consumers also rated how close the formulations were to the ideal, as assessed by the JAR tests (just about right) (1 = extremely less than ideal; 5 = extremely greater than ideal) for the attributes of color, goat aroma, herbaceous aroma, consistency, texture, salt, acidity, and herbaceous flavor [37].

### 2.11. Statistical Analysis

All assays were performed in triplicate in three different experiments, and the results were expressed as average ± standard deviation. Data were submitted to Student’s *t*-test or Analysis of Variance (ANOVA), followed by Tukey’s test, considering a significant *p*-value of <0.05. In sensory analysis, the JAR form and difference between samples (given the scale of values for each attribute) were tested using the Friedman test. Statistical analysis was performed using Sigma-Stat software, version 3.5 (Jandel Scientific Software, San Jose, CA, USA) [38].

## 3. Results

### 3.1. Technological, Physical, and Physicochemical Properties

Table 1 shows the technological parameters of goat cream cheese formulations over 21 days of refrigerated storage. The yield ranged from 189.17 to 213.46 g/L, with no significant differences between the formulations (*p* ≥ 0.05). Instrumental color revealed light yellowish-green shades, with values ranging from 77.31 to 90.72 for L*, from −2.03 to −0.75 for a*, and from 5.85 to 9.68 for b*. The addition of xique-xique flour, with or without *L. plantarum* CNPC003, notably affected instrumental color parameters (*p* < 0.05), leading to a more yellow-green appearance and decreased brightness, particularly on day 21 of storage. For the most evaluated storage times, PC had higher values for luminosity (L*) when compared to the other goat cream cheese formulations (*p* < 0.05). Supplementation with xique-xique flour contributed to reduced L* values in XC and PXC as compared to PC (*p* < 0.05). As for the a* and b* parameters, XC and PXC had lower a* and b* values when compared to PC (*p* < 0.05), where a green-yellow tone predominated probably due to the yellowish-green color of xique-xique flour.

The examined goat cream cheese formulations were overall characterized as having a soft and adhesive texture, with average values ranging from 1.30 to 3.58 N for firmness, 667.19 to 2579.14 g/s for stickiness, 0.07 to 0.96 for elasticity, 0.10 to 0.90 for cohesiveness, and 0.38 to 1.61 N for gumminess (Table 1). PC and PXC had lower firmness and adhesion and higher elasticity, cohesiveness, and gumminess when compared to XC and CC (*p* < 0.05). Most of the examined cheese formulations did not show any changes during storage regarding the measured texture parameters (*p* ≥ 0.05), except for CC, which presented an increase in adhesion and a reduction in cohesiveness (*p* < 0.05). XC had a decrease in cohesiveness and elasticity and an increase in adhesiveness (*p* < 0.05).

The physical and physicochemical parameters for the goat cream cheese formulations over 21 days of refrigerated storage are shown in Table 2. The goat cream cheese formulations had a_w_ values ranging from 0.922 to 0.925, pH ranging from 5.92 to 6.85, titratable acidity ranging from 0.25 to 0.77 g/100 g, ash contents ranging from 1.03 to 1.38 g/100 g, moisture ranging from 70.03 to 74.57 g/100 g, total protein ranging from 9.02 to 11.82 g/100 g, and fat ranging from 9.61 to 12.69 g/100 g. On day 21 of storage, a higher pH was found for PXC (*p* < 0.05). In that same evaluation time, PC, XC, and PXC had overall higher moisture contents and lower ash, protein, and fat contents than CC (*p* < 0.05), while PC and XC showed a reduction in fat content (*p* < 0.05) during storage.

### 3.2. Sugar and Organic Acid Contents

The goat cream cheese formulations had lactose (0.52–1.80 g/100 g), galactose (0.03–0.40 g/100 g), and glucose (0.17–0.80 g/100 g) as the most prevalent sugars (Table 3). The lactose contents in the goat cream cheese formulations were reduced during storage, and there were concomitant increases in galactose and glucose contents (*p* < 0.05). The lactose contents were higher in PC, XC, and PXC than in CC on day 21 of storage (*p* < 0.05). However, PXC had the lowest contents of glucose and galactose (*p* < 0.05).

The goat cream cheese formulations had lactic (0.40–1.83 g/100 g), acetic (0.01–0.04 g/100 g), and propionic acids (0.10–0.26 g/100 g) as the most prevalent organic acids (Table 3). The contents of organic acid increased, especially lactic and propionic acid, in most examined goat cream cheese formulations on day 21 of storage (*p* < 0.05), which could be linked to a higher consumption of glucose and galactose in these products over time.

### 3.3. Protein Characterization

#### 3.3.1. Determination of Soluble Protein

**Figure 2:**
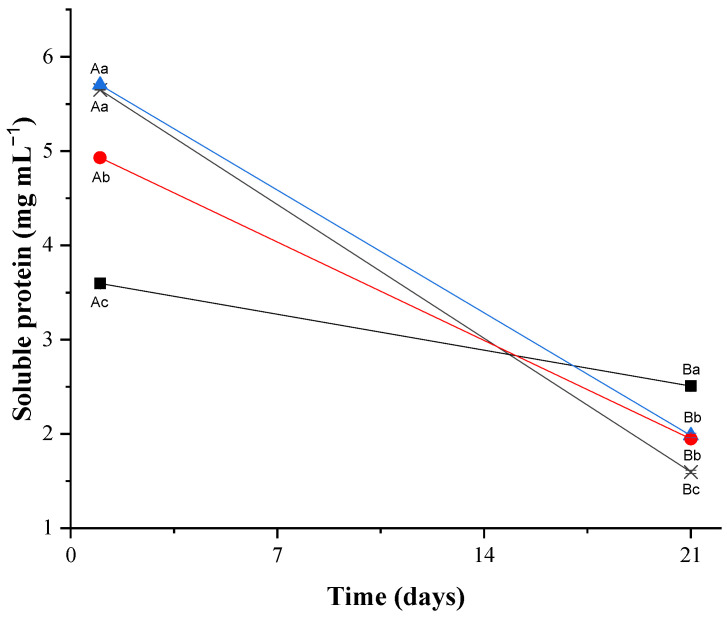
Soluble protein of goat cream cheese. Formulations: CC—goat cream cheese without Lactiplantibacillus plantarum CNPC003 and xique-xique flour (control) (**×**); PC—goat cream cheese with *L. plantarum* CNPC003 (**■**); XC—goat cream cheese with xique-xique flour (**▲**); PXC—goat cream cheese with *L. plantarum* CNPC003 and xique-xique flour (**●**). ^A,B^ Average ± standard deviation with different capital letters on same line differed based on Student’s *t*-test (*p* < 0.05) among formulations. ^a–c^ Average ± standard deviation with different lowercase letters in same column differed based on Tukey’s test (*p* < 0.05) during storage.

Figure 2 shows the decrease in the soluble protein content in cream cheeses during refrigerated storage. This reduction, observed in the examined goat cream cheese formulations, likely resulted from the processes of renin action and fermentation by lactic acid bacteria in goat milk (CC and XC) or fermentation by *L. plantarum* CNPC003 (PC and PXC).

#### 3.3.2. Electrophoretic Profile

**Figure 3:**
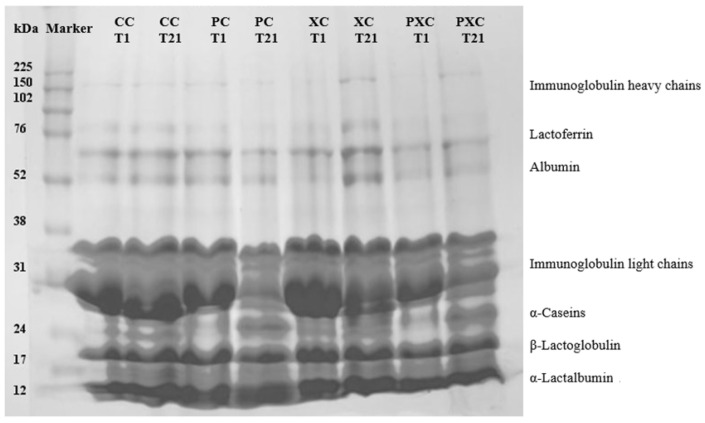
SDS-PAGE profile of goat cream cheese. Formulations: CC—goat cream cheese without *L. plantarum* CNPC003 and xique-xique flour (control); PC—goat cream cheese with *L. plantarum* CNPC003; XC—goat cream cheese with xique-xique flour; PXC—goat cream cheese with *L. plantarum* CNPC003 and xique-xique flour. T1—1 day of refrigerated storage; T21—21 days of refrigerated storage.

The protein profiles of the goat cream cheese formulations showed protein fractions ranging from 150 to 12 kDa. These fractions were identified as heavy-chain immunoglobulins (150.0 kDa), lactoferritin (76 kDa), albumin (52 kDa), light-chain immunoglobulins (31.0 kDa), β-casein (24.0 kDa), β-lactoglobulin (17.0 kDa), and α-lactalbumin (12.0 kDa) (Figure 3).

Electrophoresis in PC and PXC also showed protein hydrolysis during storage, especially albumin, likely due to higher fermentative metabolism induced by *L. plantarum* CNPC003. Extra bands corresponding to α-lactalbumin, β-lactoglobulin, and β-casein were observed in the goat cream cheese formulations. This suggests that these proteins remained undegraded during storage, possibly due to residual whey in the cheese formulations. SDS-PAGE band intensities were associated with changes in protein composition in the different goat cream cheese formulations.

#### 3.3.3. Free Amino Acid Profile

The concentration of total free amino acids (TFAAs) increased (*p* < 0.05) in all goat cream cheese formulations during storage, showing differences between the samples (Table 4). Seventeen different free amino acids (FAA) were detected in the examined cream cheese formulations, especially leucine, glutamic acid, phenylalanine, proline, and histidine, which were overall the most prevalent amino acids on day 21 of storage. Corroborating the TFAA results, there was an increase in most detected FAAs in the goat cream cheese formulations during storage, apart from aspartic acid in CC, alanine and valine in PC, and alanine and glycine in PXC, which showed reductions on day 21 of storage. Leucine was the most abundant amino acid in all examined goat cream cheese formulations on day 21 of storage.

### 3.4. Fatty Acid Profile

The goat cream cheese formulations had high contents of long-chain fatty acids, especially palmitic (C16:0) (28.21 to 33.63 g/100 g) and stearic acids (C18:0) (10.62 to 11.87 g/100 g), as well as oleic acid (C18:1 n9 cis) (16.18 to 18.86 g/100 g), a monounsaturated fatty acid (Table 5). Caproic acid (C6:0) and caprylic acid (C8:0), which are responsible for the typical aroma and flavor of goat dairy products [39], were found in small contents (1.31–1.77 and 1.93–2.29 g/100 g, respectively) in the examined goat cream cheese formulations, and they were not affected by supplementation with *L. plantarum* CNPC003 and/or xique-xique flour.

The cheese formulations also had essential fatty acids, such as omega-3 (alpha-linolenic) (0.04 to 0.13 g/100 g), omega-6 (linolenic) (2.41 to 3.21 g/100 g), gamma-linolenic (0.12 to 0.34 g/100 g), and omega-7 (palmitoleic) (0.71 to 1.08 g/100 g). Eicosapentaenoic acid (EPA) (C20:5) (1.68 to 4.67 g/100 g) and docosahexaenoic acid (DHA) (C22:6) (0.37 to 1.32 g/100 g) were also detected in the examined goat cream cheese formulations. PXC had the highest contents of DFA and polyunsaturated fatty acid (PUFA) on day 1 of storage, resulting in lower AI and HSFA values (*p* < 0.05). PXC had a lower content of palmitic acid (C16:0) on day 1 of storage, resulting in lower TI values (*p* < 0.05).

### 3.5. Profile of Volatile Compounds

Seventeen volatile compounds were identified in the examined goat cream cheese formulations, including aldehydes (1), acids (6), alcohols (4), ketones (2), terpenes (3), and hydrocarbons (1) (Table 6). The XC formulation modified the volatile compound profile compared to CC, specifically concerning the terpenes α-copaene and (+)-δ-cadinene. Conversely, the PXC formulation showed increased levels (*p* < 0.05) of acetic acid, ethylmethylacetic acid, isopentanoic acid, 2-ethylhexanol, 1-octanol, acetoin, and 1-decyne on day 21 of storage. Notably, only trans-2-decenal, an aldehyde, was detected in the PXC formulation on day 1 of storage.

The XC formulation did not affect the volatile acidic compound contents in goat cream cheese. However, the PXC formulation significantly increased the acetic acid levels on day 21 of storage (*p* < 0.05). Hexanoic acid was undetectable in PC and PXC on day 21 of storage (*p* < 0.05). On day 1 of storage, PC and PXC had lower 2-ethylhexanol levels but higher 1-octanol levels compared to XC and CC (*p* < 0.05). Additionally, PC and PXC had higher acetoin contents and lower 2-heptanone contents than XC and CC on the same day (*p* < 0.05).

On day 21 of storage, the XC formulation presented higher β-caryophyllene levels compared to PC and CC (*p* < 0.05). The terpenes α-copaene and (+)-δ-cadinene were exclusively detected in the XC formulation.

### 3.6. An Evaluation of the Hygienic–Sanitary Conditions of the Goat Cream Cheese Formulations and the Viable Cell Counts of L. plantarum CNPC003

The results of the hygienic–sanitary microbiological parameters indicated that the examined goat cream cheese formulations were suitable for human consumption over 21 days of storage, with counts lower than the minimum limits defined by current legislation (<2 log CFU/mg for coagulase-positive staphylococci and Escherichia coli and the absence of Salmonella spp.) [36].

**Figure 4:**
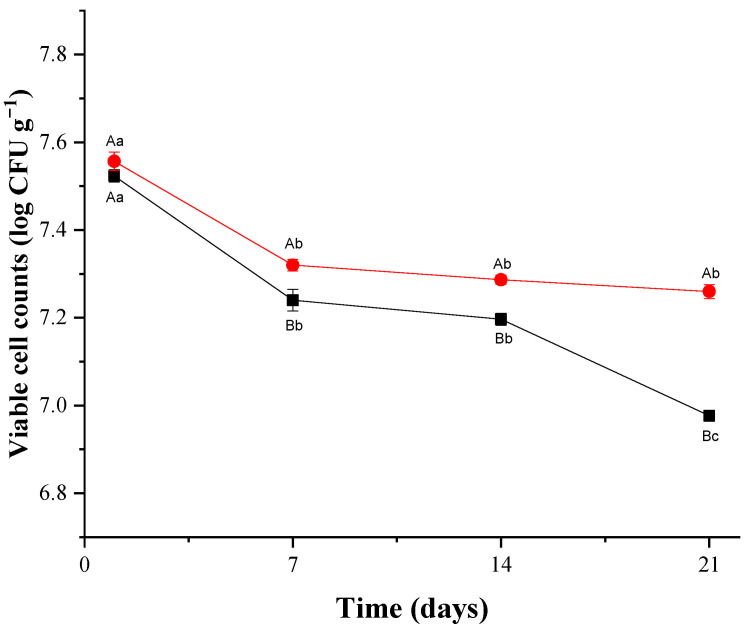
Viable cell counts of Lactiplantibacillus plantarum CNPC003 in goat cream cheese formulations during refrigerated storage. Formulations: PC—goat cream cheese with *L. plantarum* CNPC003 (**■**); PXC—goat cream cheese with *L. plantarum* CNPC003 and xique-xique flour (**●**). ^A,B^ Average ± standard deviation with different capital letters on same line differed based on Student’s *t*-test (*p* < 0.05) among formulations. ^a–c^ Average ± standard deviation with different lowercase letters in same column differed based on Tukey’s test (*p* < 0.05) during storage.

The viable cell counts of *L. plantarum* CNPC003 in the goat cream cheese formulations during storage are shown in Figure 4. The viable cell counts of *L. plantarum* CNPC003 ranged from 7.52 to 7.56 log CFU/g on day 1 of storage. The viable cell counts of *L. plantarum* CNPC003 decreased during storage in PC (7.52 ± 0.02 log CFU/g to 6.98 ± 0.02 log CFU/g) and PXC (7.56 ± 0.01 log CFU/g to 7.26 ± 0.02 log CFU/g) (*p* < 0.05). PXC had higher viable cell counts (*p* < 0.05) of *L. plantarum* CNPC003 (7.26 ± 0.01 log CFU/g) than PC (6.98 ± 0.03 log CFU/g) on day 21 of storage.

### 3.7. Sensory Analysis

**Figure 5:**
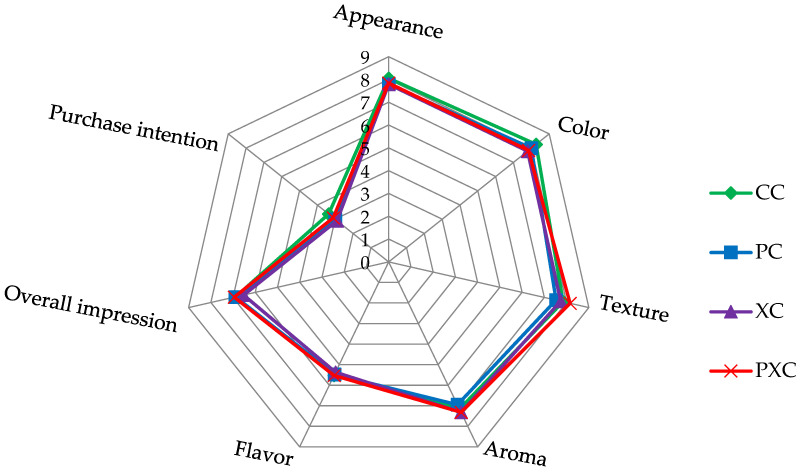
Spiderweb diagram representing acceptance test and purchase intention of goat cream cheese formulations. Formulations: CC—goat cream cheese without *Lactiplantibacillus plantarum* CNPC003 and xique-xique flour (control); PC—goat cream cheese with probiotic *L. plantarum* CNPC003; XC—goat cream cheese with xique-xique flour; PXC—goat cream cheese with probiotic *L. plantarum* CNPC003 and xique-xique flour. Acceptance test (appearance, color, texture, aroma, and flavor) on 9-point hedonic scale (1 = extremely disliked; 9 = extremely liked). Purchase intention on 5-point scale (1 = certainly would not buy; 5 = certainly would buy).

Figure 5 shows the results of the sensory acceptance and purchase intention of the examined goat cream cheese formulations. For all sensory attributes evaluated, the consumers attributed scores ranged from 5.37 to 8.26, corresponding to the hedonic terms “neither liked/nor disliked” and “liked it very much”. Generally, the average scores obtained for most of the evaluated attributes were above 5.0, demonstrating good acceptance of the examined goat cream cheese formulations. The scores assigned for purchase intention ranged from 2.89 to 3.37, corresponding to the hedonic terms “possibly would not buy” or “possibly would buy”.

The JAR results for the examined goat cream cheese formulations are shown in Table 7. On a 5-point scale, consumers were assigned scores ranging from 2.58 to 4.00, indicating that the color, goat aroma, herbaceous aroma, consistency, texture, salt, acidity, and herbaceous flavor were close to ideal. In general, for most of the evaluated attributes, supplementation with *L. plantarum* CNPC003 and/or xique-xique flour did not impact the JAR of goat cream cheese (*p* ≥ 0.05), except for the color, texture, and acidity. The darker colors of XC and PXC were considered less ideal when compared to CC (*p* < 0.05). The acidity of PC, XC, and PXC was considered closer when compared to CC (*p* < 0.05). The herbaceous aroma and flavor of PC, XC, and PXC were considered close to ideal (scores in the ranges of 3.53–3.05 and 2.74–3.58, respectively).

## 4. Discussion

In general, supplementation with xique-xique flour and *L. plantarum* CNPC003 caused changes in the technological properties of goat cream cheese, including the color and texture. The goat cream cheese formulations had reduced luminosity during storage, which could be linked to solid matrix compaction due to the formation of soluble complexes that reduce gel opacity during storage [40]. Furthermore, supplementation with xique-xique flour resulted in reduced L* values in the XC and PXC formulations compared to PC and CC. This effect can be attributed to the yellowish-green hue characteristic of xique-xique flour. Similar color tone results were observed in yogurts with xique-xique jam and in cookies made with xique-xique flour [17,18].

The soft texture of goat cheese is often related to goat milk’s composition, which has small fat globules and a low αs1-casein concentration [37]. The decreases in firmness and adhesiveness could be positive aspects of supplementation with *L. plantarum* CNPC003 since cream cheeses are expected to have a soft and non-sticky texture. A favorable effect of the use of EPS-producing probiotic bacteria has been reported on the textural and rheological characteristics of cheeses [41]. Changes in cohesiveness and elasticity in cheeses can also be attributed to proteolytic changes in protein aggregates, which are caused by the change in acidification that occurs during ripening [23].

Regarding the values of the physical and physicochemical parameters of the goat cream cheese formulations, the results agree with those previously reported for cream cheese made with cow [42] and goat milk [23]. As expected in fermented products, such as cheeses, the acidity increased and the pH decreased, and these were more expressive in PXC. The decrease in pH is a natural process resulting from cheese post-acidification and is linked to the activity of the starter and/or probiotic culture during storage, increasing lactic acid production [43]. A higher pH may also provide a more favorable environment for probiotics [44] and better consumer acceptance of fermented products [45]. In addition, the presence of xique-xique flour may have enhanced the metabolism of probiotic strains with increased exposure to acid production [16]. Another change presented was related to the fat content, with an emphasis on the PC and XC formulations that showed a reduction during storage. For food processing, a low fat content could be important from nutritional and technological points of view since lipid oxidation is one of the main problems affecting dairy product storability [46].

In this study, we observed that the addition of xique-xique and *L. plantarum* CNPC003 had distinct effects on the organic compounds and sugars in the goat cream cheese formulations. Lactose, the main sugar present in the formulations, decreased over the storage period, with increase in galactose and glucose contents, which are directly related to lactose hydrolysis by lactic acid bacteria during fermentation [47]. Regarding the differences between formulations, the lactose contents were significantly higher in the PC, XC, and PXC formulations compared to the control (CC) on day 21 of storage. This may be attributed to the increased metabolic activity of lactic acid bacteria, possibly stimulated by the addition of xique-xique, which contains prebiotic components [16]. Xique-xique may promote microorganism multiplication and enhance sugar consumption, thereby contributing to the production of organic acids, especially lactic and propionic acids, in higher amounts over time [5].

The increase in organic acids, particularly lactic and propionic acids, in the studied formulations reflects an active microbial metabolism during fermentation and storage. The higher lactic acid concentration in the xique-xique (XC and PXC) formulations is favorable from a sensory perspective, as lactic acid contributes to the characteristic flavor of fermented cheeses [48]. Additionally, the increased propionic acid concentration observed in the xique-xique formulations has interesting functional implications. Propionic acid is a short-chain fatty acid (SCFA) produced by lactic acid bacteria, including probiotic strains, and has been associated with health benefits such as anti-inflammatory and anti-diabetic effects [5,49]. The higher propionate levels in the PXC formulations may be beneficial, especially for consumers with metabolic conditions such as diabetes or obesity, as propionate production is lower in individuals with these conditions [50]. Therefore, the addition of *L. plantarum* CNPC003 and xique-xique not only altered the sugar and organic acid profiles but also had important sensory and functional implications, improving the cheese quality in terms of flavor and potential health benefits.

The decrease in the soluble protein content in cream cheeses during storage may be associated with increased proteolysis in the formulations as a result of fermentation [51]. In a previous study, *L. plantarum* CNPC003 (formerly *L. plantarum* B12) presented proteolytic capacity [10], which also could be linked to the results found in PC and PCX electrophoresis. Proteolysis in goat cream cheese can generate bioactive peptides with antioxidant, antimicrobial, immunomodulatory, and angiotensin inhibitory properties, especially when using starter and/or probiotic cultures [52,53,54,55]. These peptides are produced during dairy product processing through the action of renin peptidases and coagulants [51].

The increase in TFAAs during storage was also observed in goat milk cheese formulations, and these results are in agreement with previous studies that evaluated goat milk cheese made with different coagulants [56,57]. The production of free amino acids contributes to the characteristic flavor in cheeses in addition to providing precursors to other catabolic reactions that result in ketoacids, ammonia, amines, aldehydes, acids, and alcohols, which are key factors to cheese’s taste and aroma [58]. Leucine, valine, phenylalanine, tyrosine, and methionine are the main precursors of aromatic compounds in cheese [59], and these amino acids were found in the highest concentrations in XC and PXC on day 21 of storage. It is worth noting that the proteolytic processes of the action of proteinases and peptidases produced by probiotic strains can generate free amino acids that, together with other biochemical reactions (intensified by the presence of prebiotics), cause the transformation into compounds such as esters, volatile compounds, and others [41]. These transformations significantly impact the sensory characteristics, shelf life, and potential health benefits of the product, making it more appealing and functional for consumers.

Supplementation with *L. plantarum* CNPC003 and xique-xique flour improved the fatty acid profile of the goat cream cheeses, with a positive impact on lipid quality indices. The goat cream cheese formulations had high contents of long-chain fatty acids. Long-chain fatty acids are associated with the increased adhesion of probiotic cultures to the distal intestinal mucosal layer [60]. The cheese formulations also had essential fatty acids, which are associated with a reduced chronic disease risk [61]. The consumption of these fatty acids is associated with decreased blood cholesterol levels and cardiovascular disease risk [62].

The number of volatile compounds identified in the examined goat cream cheese formulations was lower compared to a prior study on goat cheese [48]. This may be related to the fact that the goat cream cheese formulations examined in this study were fresh and had a short storage time, with a low impact on the aromatic profile, especially when compared to ripened cheeses. This is the first study focusing on the volatile profile of goat cream cheese, and the detected compounds could better represent the volatile profile of this goat dairy product. The increased production of volatile compounds over time could be caused by biochemical reactions during storage. Therefore, the longer the storage period, the more intense the reactions, with an increased production of volatile compounds [48].

Aldehydes were detected only in the PXC formulation on day 1 of storage. Aldehydes are typically transient compounds, reaching high concentrations immediately after processing, but they are rapidly converted into their corresponding alcohols and acids [48]. These compounds play a crucial role in the aroma and flavor profile of food products [63], and trans-2-decenal is specifically associated with a fatty aroma in dairy products [64].

The exclusive presence of trans-2-decenal in the PXC formulation may be linked to the synergistic effects of *L. plantarum* CNPC003 and xique-xique flour. Lactic acid bacteria, particularly *L. plantarum*, are known to produce aldehydes during fermentation as part of their metabolic pathways, and the addition of xique-xique, which contains bioactive components, may further influence the microbial metabolism and the subsequent formation of such volatile compounds [65]. The combination of these two ingredients may have promoted a unique metabolic environment, leading to the presence of trans-2-decenal in this formulation. Acetic acid was the most abundant organic acid in the examined goat cream cheese formulations. Acetic acid accounts for the fatty/cheese flavor in fermented dairy products [63]. Hexanoic acid, not detected in PC and PXC, is associated with the typical aroma of goat milk and the cloying, sweet, and rancid aromas of cheese [6]. Therefore, reductions in hexanoic acid contents are important to reduce the goat aroma in goat dairy products.

The detection of α-copaene and (+)-δ-cadinene only in the XC formulation may be related to the presence of these compounds in xique-xique flour. Cactus plants, such as those from the Caatinga, have developed unique adaptations to survive in harsh environmental conditions, which include the synthesis of various phytochemicals, such as phenolic acids, alkaloids, flavonoids, terpenes, and tannins. These compounds are part of the plant’s defense mechanisms and possess notable biological activities [66,67]. The presence of α-copaene and (+)-δ-cadinene in XC but not in PXC may be explained by the specific role of xique-xique flour. When added alone, xique-xique flour contributes these terpenoid compounds, which are part of the plant’s essential oils and are known for their aroma and biological functions. However, in the PXC formulation, the presence of *L. plantarum* CNPC003, a lactic acid bacterium, may have altered the microbial metabolism, potentially degrading or modifying the terpenes present in the flour. Lactic acid bacteria, including *L. plantarum*, are known to metabolize various compounds during fermentation, and this could have led to the reduction or transformation of terpenoid compounds like α-copaene and (+)-δ-cadinene [68].

Regarding the *L. plantarum* CNPC003 count, the PXC formulation had higher viable cell counts than PC on day 21 of storage. This characteristic could be attributed to a possible prebiotic effect promoted by xique-xique flour primarily linked to its total fiber content (16.59 ± 0.09 g/100 g) [21]. The prebiotic potential of lyophilized xique-xique juice was previously reported, with selective stimulatory effects on the growth and metabolism of distinct probiotic lactobacilli compared to fructooligosaccharide—FOS (a proven prebiotic ingredient) [16]. PC and PXC had viable cell counts of *L. plantarum* CNPC003 above the commonly recommended minimum count (i.e., 6 to 7 log CFU/g) that causes beneficial health effects on the host [69]. This result suggests that *L. plantarum* CNPC003 kept a high survival rate in goat cream cheese under refrigerated storage, indicating that xique-xique flour could not negatively affect the survival of this potentially probiotic strain in goat cream cheese until consumption [3,5].

Supplementation with *L. plantarum* CNPC003 and/or xique-xique flour did not negatively impact the sensory acceptance of the goat cream cheese formulations, except for the color and texture. These data indicate that changes in instrumental color, organic acid and sugar contents, and instrumental texture in the examined goat cream cheese formulations did not negatively affect consumer acceptance. The distinct flavor of goat milk likely influenced the lower flavor scores attributed to the examined goat cream cheese formulations. However, the perception of “goat flavor” did not affect the overall acceptance of the goat cream cheese formulations that received scores above 6 (“liked slightly” to “liked moderately”). The data demonstrate that supplementation with plant components could be an option to help overcome common difficulties in marketing goat dairy products due to their distinct flavor characteristics perceived by some consumers [17,37,70].

## 5. Conclusions

This study represents the first evaluation of the impact of supplementing goat cream cheese with the potentially probiotic strain *L. plantarum* CNPC003 and xique-xique flour, focusing on technological, nutritional, physicochemical, and sensory properties. The results show that the addition of xique-xique flour contributed to a distinct yellowish-green hue in the cheese and influenced the texture, fatty acid profile, and volatile compound composition. Importantly, the inclusion of *L. plantarum* CNPC003 promoted probiotic viability, with the strain achieving >7 log CFU/g in cheese containing xique-xique flour after 21 days of storage, underscoring its potential as a viable probiotic ingredient. Combined supplementation with *L. plantarum* CNPC003 and xique-xique flour also enhanced the nutritional profile by increasing the contents of DFA, PUFA, and bioactive compounds while reducing undesirable saturated fatty acids. Although supplementing the cheese formulations with *L. plantarum* CNPC003 and xique-xique flour impacted the color and texture, it did not negatively affect consumer acceptance. This study highlights the promising potential of combining supplementation with *L. plantarum* CNPC003 and xique-xique flour in goat cream cheese production, not only to improve product quality but also to enhance its functional properties, paving the way for the further exploration and commercialization of these ingredients in the dairy industry.

## Figures and Tables

**Table 1:** Yields, color parameters, and texture profiles of goat cream cheese over 21 days of refrigerated storage.

Parameters	Days of Storage	Formulations			
		CC	PC	XC	PXC
Yield (g/L)	1	213.46 ± 32.23 ^A^	208.34 ± 19.33 ^A^	195.70 ± 57.22 ^A^	189.17 ± 46.44 ^A^
L*	1	83.31 ± 0.06 ^Db^	90.72 ± 0.19 ^Aa^	84.75 ± 0.32 ^Ca^	85.84 ± 0.05 ^Ba^
	7	85.04 ± 0.04 ^Ba^	86.21 ± 0.22 ^Ab^	81.71 ± 0.16 ^Db^	83.89 ± 0.09 ^Cb^
	14	81.75 ± 0.05 ^Bc^	85.61 ± 0.15 ^Ac^	78.03 ± 0.01 ^Dc^	79.72 ± 0.30 ^Cc^
	21	81.39 ± 0.19 ^Ac^	81.99 ± 0.19 ^Ad^	77.31 ± 0.19 ^Cd^	79.91 ± 0.11 ^Bc^
a*	1	−1.83 ± 0.04 ^Ba^	−2.01 ± 0.02 ^Aa^	−1.99 ± 0.02 ^Aa^	−1.54 ± 0.03 ^Ca^
	7	−0.82 ± 0.02 ^Db^	−1.18 ± 0.01 ^Cc^	−1.85 ± 0.01 ^Ab^	−1.41 ± 0.02 ^Bb^
	14	−0.75 ± 0.02 ^Dc^	−1.27 ± 0.01 ^Cb^	−2.03 ± 0.04 ^Aa^	−1.36 ± 0.01 ^Bc^
	21	−0.75 ± 0.01 ^Dc^	−1.16 ± 0.01 ^Bc^	−1.81 ± 0.02 ^Ab^	−0.96 ± 0.01 ^Cd^
b*	1	5.85 ± 0.01 ^Cd^	7.48 ± 0.07 ^Bb^	9.08 ± 0.07 ^Ab^	7.46 ± 0.04 ^Bd^
	7	6.86 ± 0.01 ^Cc^	7.91 ± 0.02 ^Ba^	9.66 ± 0.07 ^Aa^	7.73 ± 0.08 ^Bc^
	14	6.47± 0.02 ^Db^	7.95 ± 0.06 ^Ca^	9.68 ± 0.09 ^Aa^	8.43 ± 0.09 ^Bb^
	21	7.43 ± 0.08 ^Ca^	7.43 ± 0.03 ^Cb^	8.39 ± 0.06 ^Bc^	9.22 ± 0.04 ^Aa^
Hardness (N)	1	3.58 ± 0.60 ^Aa^	1.85 ± 0.53 ^Ba^	2.75 ± 0.18^A Ba^	1.76 ± 0.25 ^Ba^
	7	2.95 ± 0.45 ^Aa^	1.70 ± 0.32 ^Ba^	2.66 ± 0.27 ^Aa^	1.41 ± 0.20 ^Ba^
	14	2.57 ± 0.15 ^Aa^	1.45 ± 0.30 ^Ba^	2.52 ± 0.35 ^Aa^	1.30 ± 0.08 ^Ba^
	21	3.15 ± 0.69 ^Aa^	1.38 ± 0.05 ^Ba^	3.40 ± 0.31 ^Aa^	1.41 ± 0.20 ^Ba^
Adhesiveness (g/s)	1	2290.95 ± 112 ^Ab^	781.39 ± 105 ^Ba^	2145.78 ± 165 ^Ab^	870.05 ± 87.7 ^Ba^
	7	2527.63 ± 70.5 ^Aa^	748.65 ± 66.3 ^Ba^	2167.48 ± 100 ^Ab^	787.53 ± 61.2 ^Ba^
	14	1839.97 ± 415 ^Ab^	729.51 ± 14.8 ^Ba^	2242.26 ± 18.3 ^Aab^	667.19 ± 46.6 ^Ba^
	21	2579.14 ± 33.5 ^Aa^	715.46 ± 27.1 ^Ba^	2515.34 ± 44.1 ^Aa^	728.05 ± 96.6 ^Ba^
Springiness	1	0.19 ± 0.04 ^Ba^	0.93 ± 0.01 ^Aa^	0.34 ± 0.15 ^Ba^	0.94 ± 0.01 ^Aa^
	7	0.13 ± 0.03 ^Ba^	0.70 ± 0.28 ^Aa^	0.21 ± 0.04 ^Ba^	0.94 ± 0.02 ^Aa^
	14	0.13 ± 0.03 ^Ba^	0.93 ± 0.01 ^Aa^	0.18 ± 0.05 ^Bab^	0.94 ± 0.01 ^Aa^
	21	0.07 ± 0.02 ^Ba^	0.95 ± 0.01 ^Aa^	0.13 ± 0.03 ^Bb^	0.96 ± 0.01 ^Aa^
Cohesiveness	1	0.24 ± 0.03 ^Ba^	0.87 ± 0.01 ^Aa^	0.32 ± 0.10 ^Bab^	0.87 ± 0.01 ^Aa^
	7	0.22 ± 0.01 ^Ca^	0.87 ± 0.01 ^Aa^	0.35 ± 0.07 ^Ba^	0.82 ± 0.01 ^Aa^
	14	0.18 ± 0.03 ^Ca^	0.88 ± 0.01 ^Aa^	0.25 ± 0.01 ^Bb^	0.89 ± 0.01 ^Aa^
	21	0.10 ± 0.03 ^Bb^	0.90 ± 0.01 ^Aa^	0.15 ± 0.04 ^Bc^	0.88 ± 0.01 ^Aa^
Gumminess (N)	1	0.77 ± 0.28 ^Aa^	1.61 ± 0.45 ^Aa^	1.02 ± 0.41 ^Aa^	1.53 ± 0.21 ^Aa^
	7	0.75 ± 0.15 ^Ba^	1.24 ± 0.03 ^Aa^	0.57 ± 0.19 ^Ba^	1.49 ± 0.27 ^Aa^
	14	0.82± 0.23^ABa^	1.28 ± 0.27 ^Aa^	0.63 ± 0.16 ^Ba^	1.16 ± 0.07^ABa^
	21	0.38 ± 0.05 ^Ba^	1.34 ± 0.05 ^Aa^	0.52 ± 0.08 ^Ba^	1.23 ± 0.18 ^Aa^

Results are expressed as average (*n* = 9) ± standard deviation. L* ranges from 0 (black) to 100 (white), a* ranges from green (−a*) to red (+a*), and b* ranges from blue (−b*) to yellow (+b*). Formulations: CC—goat cream cheese without probiotic and xique-xique flour (control); PC—goat cream cheese with addition of probiotic *Lactiplantibacillus plantarum* CNPC003; XC—goat cream cheese with addition of xique-xique flour; PXC—goat cream cheese with addition of probiotic *Lactiplantibacillus plantarum* CNPC003 and xique-xique flour. ^A–D^ Average ± standard deviation with different capital letters on same line differed based on Tukey’s test (*p* < 0.05) among formulations. ^a–d^ Average ± standard deviation with different lowercase letters in same column differed based on Tukey’s test (*p* < 0.05) during storage.

**Table 2:** Chemical compositions and physicochemical characteristics of goat cream cheese over 21 days of refrigerated storage.

Parameters	Days of Storage	Formulations			
		CC	PC	XC	PXC
a_w_ ^1^	1	0.924 ± 0.001 ^Aa^	0.922 ± 0.001 ^Cb^	0.923 ± 0.001 ^Ba^	0.924 ± 0.001 ^Ab^
	7	0.923 ± 0.001 ^Cb^	0.923 ± 0.001 ^Ca^	0.922 ± 0.001 ^Bb^	0.924 ± 0.001 ^Ab^
	14	0.923 ± 0.001 ^Bb^	0.923 ± 0.001 ^Ba^	0.923 ± 0.001 ^Ba^	0.925 ± 0.001 ^Aa^
	21	0.922 ± 0.001 ^Cc^	0.923 ± 0.001 ^Ba^	0.923 ± 0.001 ^Ba^	0.925 ± 0.001 ^Aa^
pH	1	6.75 ± 0.01 ^Da^	6.83 ± 0.01 ^Ba^	6.85 ± 0.00 ^Aa^	6.81 ± 0.01 ^Ca^
	7	6.45 ± 0.00 ^Db^	6.47 ± 0.00 ^Cb^	6.74 ± 0.01 ^Ab^	6.58 ± 0.01 ^Bb^
	14	6.19 ± 0.01 ^Dc^	6.46 ± 0.01 ^Bb^	6.41 ± 0.01 ^Cc^	6.60 ± 0.00 ^Ab^
	21	5.92 ± 0.00 ^Dd^	6.20 ± 0.01 ^Cc^	6.31 ± 0.01 ^Bd^	6.41 ± 0.01 ^Ac^
Lactic acid acidity (g/100 g)	1	0.25 ± 0.01 ^Ad^	0.25 ± 0.02 ^Ad^	0.25 ± 0.01 ^Ad^	0.26 ± 0.01 ^Ad^
	7	0.37 ± 0.01 ^Cc^	0.36 ± 0.01 ^Cc^	0.42 ± 0.01 ^Bc^	0.52 ± 0.01 ^Ac^
	14	0.42 ± 0.01 ^Cb^	0.43 ± 0.01 ^Cb^	0.52 ± 0.01 ^Bb^	0.68 ± 0.01 ^Ab^
	21	0.52 ± 0.01 ^Da^	0.69 ± 0.01 ^Ba^	0.61 ± 0.02 ^Ca^	0.77 ± 0.01 ^Aa^
Ash (g/100 g)	1	1.37 ± 0.14 ^Aa^	1.03 ± 0.04 ^Ba^	1.23 ± 0.05 ^ABa^	1.13 ± 0.03 ^Ba^
	7	1.32 ± 0.04 ^Aa^	1.14 ± 0.15 ^Aa^	1.23 ± 0.06 ^Aa^	1.22 ± 0.05 ^Aa^
	14	1.38 ± 0.06 ^Aa^	1.13 ± 0.09 ^Ba^	1.25 ± 0.06 ^ABa^	1.25 ± 0.04 ^ABa^
	21	1.33 ± 0.04 ^Aa^	1.12 ± 0.08 ^Ba^	1.24 ± 0.04 ^ABa^	1.10 ± 0.10 ^Ba^
Moisture (g/100 g)	1	71.56 ± 0.60 ^Ba^	74.57 ± 0.41 ^Aa^	72.42 ± 0.52 ^Ba^	72.37 ± 0.30 ^Ba^
	7	70.90 ± 0.62 ^Cab^	74.09 ± 0.15 ^Aab^	72.20 ± 0.39 ^BCa^	72.48 ± 0.53 ^Ba^
	14	70.11 ± 0.32 ^Bab^	72.60 ± 0.19 ^Ab^	72.31 ± 0.35 ^Aa^	72.22 ± 0.52 ^Aa^
	21	70.03 ± 0.34 ^Bb^	72.25 ± 0.15 ^Ab^	71.76 ± 0.42 ^Aa^	71.63 ± 0.47 ^Aa^
Protein (g/100 g)	1	11.82 ± 0.01 ^Aa^	10.26 ± 0.35 ^Ba^	11.32 ± 0.14 ^Aa^	10.52 ± 0.42 ^Ba^
	7	11.73 ± 0.16 ^Aa^	9.66 ± 0.12 ^Ba^	11.73 ± 0.22 ^Aa^	10.15 ± 0.43 ^Ba^
	14	11.08 ± 0.05 ^Ab^	9.69 ± 0.19 ^Ba^	11.36 ± 0.06 ^Aa^	9.20 ± 0.12 ^Cb^
	21	10.66 ± 0.20 ^Ab^	9.02 ± 0.37 ^Bb^	10.37 ± 0.21 ^Ab^	9.20 ± 0.16 ^Bb^
Fat (g/100 g)	1	12.69 ± 0.39 ^Aa^	11.45 ± 0.06 ^Bb^	11.70 ± 0.42 ^Ab^	10.45 ± 0.33 ^Ca^
	7	11.33 ± 0.20 ^Aa^	11.65 ± 0.26 ^Aa^	11.84 ± 0.30 ^Aa^	10.94 ± 0.11 ^Aa^
	14	10.66 ± 0.13 ^Aa^	10.14 ± 0.16 ^Ac^	10.40 ± 0.52 ^Ac^	10.48 ± 0.21 ^Aa^
	21	10.79 ± 0.35 ^Aa^	9.61 ± 0.34 ^Bc^	10.04 ± 0.14 ^Bc^	10.20 ± 0.11 ^Ba^

Results are expressed as average (*n* = 9) ± standard deviation. ^1^ a_w_—water activity. Formulations: CC—goat cream cheese without probiotic and xique-xique flour (control); PC—goat cream cheese with addition of probiotic *Lactiplantibacillus plantarum* CNPC003; XC—goat cream cheese with addition of xique-xique flour; PXC—goat cream cheese with addition of probiotic *Lactiplantibacillus plantarum* CNPC003 and xique-xique flour. ^A–D^ Average ± standard deviation with different capital letters on same line differed based on Tukey’s test (*p* < 0.05) between cheese formulations. ^a–d^ Average ± standard deviation with different lowercase letters in same column differed based on Tukey’s test (*p* < 0.05) during storage.

**Table 3:** Sugar and organic acid profiles of goat cream cheese on days 1 and 21 of refrigerated storage.

Parameters	Days of Storage	Formulations			
		CC	PC	XC	PXC
Sugar (g/100 g)					
Lactose	1	1.80 ± 0.13 ^Aa^	1.56 ± 0.02 ^Ba^	1.60 ± 0.03 ^Ba^	1.60 ± 0.11 ^Ba^
	21	0.52 ± 0.01 ^Cb^	0.98 ± 0.02 ^Ab^	0.93 ± 0.02 ^Ab^	0.83 ± 0.02 ^Bb^
Galactose	1	0.03 ± 0.01 ^Ab^	nd	nd	nd
	21	0.39 ± 0.10 ^Aa^	0.11 ± 0.01 ^Ba^	0.40 ± 0.01 ^Aa^	nd
Glucose	1	0.17 ± 0.01 ^Bb^	0.49 ± 0.04 ^Ab^	0.23 ± 0.01 ^Bb^	0.19 ± 0.01 ^Bb^
	21	0.40 ± 0.04 ^Ca^	0.80 ± 0.01 ^Aa^	0.61 ± 0.02 ^Ba^	0.32 ± 0.01 ^Ca^
Organic acids (g/100 g)					
Lactic	1	0.40 ± 0.13 ^Ab^	0.61 ± 0.11 ^Aa^	0.68 ± 0.15 ^Ab^	0.68 ± 0.08 ^Ab^
	21	1.43 ± 0.12 ^Aa^	0.93 ± 0.20 ^Ba^	1.83 ± 0.35 ^Aa^	1.25 ± 0.18 ^ABa^
Acetic	1	0.01 ± 0.01 ^Bb^	0.02 ± 0.01 ^Aa^	0.01 ± 0.01 ^Bb^	0.02 ± 0.01 ^Aa^
	21	0.04 ± 0.01 ^Aa^	0.03 ± 0.01 ^ABa^	0.04 ± 0.01 ^Aa^	0.02 ± 0.01 ^Ba^
Propionic	1	0.13 ± 0.01 ^Ab^	0.12 ± 0.01 ^Ab^	0.11 ± 0.04 ^Ab^	0.10 ± 0.02 ^Aa^
	21	0.26 ± 0.01 ^Aa^	0.19 ± 0.01 ^ABa^	0.24 ± 0.03 ^Aa^	0.13 ± 0.02 ^Ba^

Results are expressed as average (*n* = 9) ± standard deviation. Sugars and organic acids were quantified through equation of straight line constructed from injection of standards. Abbreviations: nd—not detected. Formulations: CC—goat cream cheese without probiotic (control); PC—goat cream cheese with addition of probiotic *Lactiplantibacillus plantarum* CNPC003; XC—goat cream cheese with addition of xique-xique flour; PXC—goat cream cheese with addition of probiotic *Lactiplantibacillus plantarum* CNPC003 and xique-xique flour. ^A–C^ Average ± standard deviation with different capital letters on same line differed based on Tukey’s test (*p* < 0.05) among cheese formulations. ^a,b^ Average ± standard deviation with different lowercase letters in same column differed based on Student’s *t*-test (*p* < 0.05) during storage.

**Table 4:** Concentrations of free amino acids (FAAs) of creamy goat cheese on days 1 and 21 of refrigerated storage.

Amino Acids (mg/100)	Days of Storage	Formulations			
		CC	PC	XC	PXC
Aspartic acid	1	2.97 ± 0.44 ^Aa^	1.52 ± 0.27 ^Bb^	1.09 ± 0.25 ^Cb^	0.17 ± 0.10 ^Cb^
	21	0.24 ± 0.03 ^Db^	13.78 ± 0.25 ^Aa^	2.21 ± 0.10 ^Ca^	8.41 ± 0.66 ^Ba^
Glutamic acid	1	0.32 ± 0.37 ^Cb^	18.86 ± 1.58 ^Bb^	0.70 ± 0.18 ^Cb^	42.78 ± 2.43 ^Ab^
	21	7.42 ± 0.15 ^Da^	66.34 ± 0.61 ^Ba^	30.51 ± 0.13 ^Ca^	100.65 ± 5.55 ^Aa^
Serine	1	1.00 ± 0.02 ^Ba^	1.84 ± 0.02 ^Aa^	2.08 ± 0.55 ^Ab^	0.44 ± 0.03 ^Ba^
	21	1.44 ± 0.40 ^Ba^	2.31 ± 0.67 ^Ba^	6.89 ± 0.29 ^Aa^	0.99 ± 0.23 ^Ba^
Glycine	1	6.77 ± 0.16 ^Aa^	6.61 ± 0.11 ^Ab^	6.23 ± 0.02 ^Ba^	7.74 ± 0.17 ^Ca^
	21	7.08 ± 0.19 ^Ba^	9.79 ± 0.75 ^Aa^	6.38 ± 0.05 ^Ba^	5.40 ± 0.27 ^Bb^
Histidine	1	18.42 ± 0.58 ^Aa^	18.79 ± 0.06 ^Ab^	18.17 ± 0.09 ^Aa^	14.76 ± 0.58 ^Ba^
	21	17.75 ± 0.06 ^Ba^	28.73 ± 0.53 ^Aa^	19.51 ± 0.32 ^Ba^	15.24 ± 1.76 ^Ca^
Arginine	1	1.35 ± 0.02 ^Cb^	1.27 ± 0.05 ^Cb^	1.60 ± 0.05 ^Bb^	1.93 ± 0.16 ^Ab^
	21	11.47 ± 0.25 ^Ba^	15.57 ± 1.12 ^Aa^	5.88 ± 0.09 ^Da^	8.68 ± 0.41 ^Ca^
Threonine	1	0.01 ± 0.01 ^Cb^	7.92 ± 0.46 ^Ab^	5.29 ± 0.79 ^Bb^	5.13 ± 0.15 ^Ba^
	21	5.90 ± 0.04 ^Ca^	13.38 ± 2.19 ^Aa^	8.44 ± 0.07 ^Ba^	5.28 ± 0.02 ^Ca^
Alanine	1	4.29 ± 0.13 ^Cb^	8.65 ± 0.55 ^Aa^	4.04 ± 0.06 ^Cb^	5.40 ± 0.05 ^Ba^
	21	19.00 ± 0.03 ^Aa^	1.45 ± 0.15 ^Db^	15.62 ± 0.01 ^Ba^	3.72 ± 0.16 ^Cb^
Proline	1	22.62 ± 0.25 ^Ba^	28.30 ± 0.05 ^Ab^	20.72 ± 1.11 ^Cb^	23.31 ± 0.52 ^Bb^
	21	21.37 ± 0.67 ^Ca^	36.19 ± 0.47 ^Ba^	48.05 ± 1.57 ^Aa^	46.87 ± 1.49 ^Aa^
Tyrosine	1	4.42 ± 0.33 ^Ba^	6.22 ± 0.08 ^Ab^	3.88 ± 0.23 ^Cb^	4.57 ± 0.18 ^Bb^
	21	4.49 ± 0.35 ^Ca^	10.54 ± 0.08 ^Aa^	4.48 ± 0.29 ^Ca^	9.57 ± 0.04 ^Ba^
Valine	1	2.84 ± 0.08 ^Cb^	8.07 ± 0.51 ^Aa^	1.47 ± 0.09 ^Db^	5.22 ± 0.27 ^Bb^
	21	9.86 ± 0.12 ^Ca^	1.31 ± 0.09 ^Db^	20.69 ± 0.28 ^Aa^	15.67 ± 1.02 ^Ba^
Methionine	1	0.22 ± 0.04 ^Aa^	0.46 ± 0.09 ^Ab^	0.50 ± 0.23 ^Ab^	0.07 ± 0.08 ^Ba^
	21	1.59 ± 1.64 ^Ba^	3.01 ± 0.47 ^Ba^	12.27 ± 1.12 ^Aa^	1.48 ± 1.05 ^Ba^
Cysteine	1	4.08 ± 0.10 ^Aa^	2.59 ± 0.02 ^Bb^	3.96 ± 0.06 ^Ab^	1.21 ± 0.67 ^Ca^
	21	3.46 ± 1.01 ^Ca^	9.13 ± 0.60 ^Aa^	7.33 ± 0.10 ^Ba^	2.13 ± 0.34 ^Ca^
Isoleucine	1	0.74 ± 0.24 ^Cb^	3.86 ± 0.25 ^Ab^	1.15 ± 0.26 ^Cb^	2.31 ± 0.08 ^Bb^
	21	4.09 ± 0.86 ^Ca^	9.59 ± 1.24 ^Ba^	11.42 ± 0.75^A Ba^	13.29 ± 0.56 ^Aa^
Leucine	1	0.90 ± 0.32 ^Cb^	23.48 ± 0.56 ^Ab^	0.80 ± 0.01 ^Cb^	14.37 ± 1.83 ^Bb^
	21	28.53 ± 0.23 ^Ca^	125.63 ± 0.49 ^Aa^	91.77 ± 0.24 ^Ba^	87.02 ± 4.34 ^Ba^
Phenylalanine	1	3.67 ± 0.22 ^Bb^	3.50 ± 0.49 ^Bb^	2.72 ± 0.15 ^Bb^	15.88 ± 0.39 ^Ab^
	21	29.57 ± 0.51 ^Ca^	22.28 ± 0.37 ^Ca^	57.70 ± 1.47 ^Ba^	71.35 ± 4.17 ^Aa^
Lysine	1	1.34 ± 0.24 ^Ba^	11.90 ± 0.54 ^Aa^	1.88 ± 0.83 ^Ba^	15.02 ± 3.56 ^Aa^
	21	1.81 ± 0.07 ^Ca^	7.85 ± 2.81 ^Ba^	4.03 ± 0.21 ^Ba^	14.18 ± 2.23 ^Aa^
TFAA	1	75.95 ± 0.95 ^Bb^	153.85 ± 3.24 ^Ab^	76.27 ± 1.55 ^Bb^	157.35 ± 0.72 ^Ab^
	21	175.06 ± 1.28 ^Da^	372.89 ± 2.13 ^Ba^	353.18 ± 6.37 ^Ca^	409.95 ± 19.2 ^Aa^

Results are expressed as average (*n* = 9) ± standard deviation. Abbreviation: TFAA—total free amino acids. Formulations: CC—goat cream cheese without probiotic and xique-xique flour (control); PC—goat cream cheese with probiotic *Lactiplantibacillus plantarum* CNPC003; XC—goat cream cheese with xique-xique flour; PXC—goat cream cheese with probiotic *Lactiplantibacillus plantarum* CNPC003 and xique-xique flour. ^A–D^ Average ± standard deviation with different capital letters on same line differed based on Tukey’s test (*p* < 0.05) among cheese formulations. ^a,b^ Average ± standard deviation with different lowercase letters in same column differed based on Student’s *t*-test (*p* < 0.05) during storage.

**Table 5:** Fatty acid profiles of goat cream cheese on days 1 and 21 of refrigerated storage.

Fatty Acids	Days of Storage	Formulations			
		CC	PC	XC	PXC
Short-chain fatty acids (g/100 g)					
Butyric (C4:0)	1	0.95 ± 0.07 ^Aa^	1.00 ± 0.05 ^Aa^	0.91 ± 0.01 ^Aa^	0.92 ± 0.03 ^Aa^
	21	0.97 ± 0.14 ^Aa^	0.82 ± 0.08 ^Ab^	0.79 ± 0.05 ^Aa^	1.00 ± 0.16 ^Aa^
Medium chain					
Caproic (C6:0)	1	1.54 ± 0.00 ^Aa^	1.60 ± 0.10 ^Aa^	1.31 ± 0.34 ^Aa^	1.68 ± 0.08 ^Aa^
	21	1.57 ± 0.20 ^Aa^	1.46 ± 0.13 ^Aa^	1.52 ± 0.17 ^Aa^	1.77 ± 0.28 ^Aa^
Caprylic (C8:0)	1	2.08 ± 0.06 ^Aa^	2.20 ± 0.13 ^Aa^	2.29 ± 0.14 ^Aa^	2.25 ± 0.07 ^Aa^
	21	2.18 ± 0.20 ^Aa^	2.00 ± 0.14 ^Aa^	1.93 ± 0.05 ^Ab^	2.05 ± 0.05 ^Aa^
Capric (C10:0)	1	7.93 ± 0.32 ^Aa^	8.20 ± 0.17 ^Aa^	7.92 ± 0.44 ^Aa^	8.31 ± 0.01 ^Aa^
	21	7.91 ± 0.10 ^Ba^	8.11 ± 0.08 ^Ba^	7.61 ± 0.14 ^Ca^	8.49 ± 0.09 ^Aa^
Undecylic (C11:0)	1	0.27 ± 0.06 ^Aa^	0.28 ± 0.04 ^Aa^	0.28 ± 0.08 ^Aa^	0.28 ± 0.02 ^Aa^
	21	0.25 ± 0.01 ^ABa^	0.25 ± 0.02 ^ABa^	0.22 ± 0.01 ^Ba^	0.28 ± 0.00 ^Aa^
Lauric (C12:0)	1	3.39 ± 0.01 ^Aa^	3.44 ± 0.14 ^Aa^	3.06 ± 0.48 ^Aa^	3.41 ± 0.04 ^Aa^
	21	3.54 ± 0.20 ^Aa^	3.19 ± 0.27 ^Aa^	3.25 ± 0.04 ^Aa^	3.67 ± 0.04 ^Aa^
Tridecylic (C13:0)	1	0.16 ± 0.01 ^Aa^	0.13 ± 0.01 ^Ba^	0.15 ± 0.01 ^Aa^	0.13 ± 0.00 ^Bb^
	21	0.13 ± 0.01 ^Bb^	0.12 ± 0.01 ^Ba^	0.12 ± 0.01 ^Ba^	0.16 ± 0.02 ^Aa^
Myristic (C14:0)	1	9.59 ± 0.08 ^Aa^	9.63 ± 0.29 ^Aa^	9.48 ± 0.19 ^Aa^	9.40 ± 0.04 ^Aa^
	21	10.17 ± 1.28 ^Aa^	9.59 ± 0.09 ^Aa^	9.32 ± 0.08 ^Aa^	9.89 ± 0.34 ^Aa^
Myristoleic (C14:1)	1	0.44 ± 0.01 ^Aa^	0.41 ± 0.02 ^Aa^	0.40 ± 0.09 ^Aa^	0.37 ± 0.03 ^Aa^
	21	0.45 ± 0.09 ^Aa^	0.34 ± 0.04 ^Aa^	0.39 ± 0.00 ^Aa^	0.40 ± 0.02 ^Aa^
Pentadecylic (C15:0)	1	1.02 ± 0.03 ^Aa^	0.94 ± 0.05 ^Aa^	1.00 ± 0.05 ^Aa^	0.94 ± 0.02 ^Aa^
	21	1.01 ± 0.17 ^Aa^	0.84 ± 0.04 ^Aa^	0.90 ± 0.00 ^Aa^	0.82 ± 0.06 ^Aa^
Pentadecenoic (C15:1)	1	0.29 ± 0.04 ^Aa^	0.22 ± 0.01 ^Aa^	0.24 ± 0.08 ^Aa^	0.24 ± 0.02 ^Aa^
	21	0.33 ± 0.04 ^Aa^	0.20 ± 0.01 ^Ba^	0.24 ± 0.05 ^ABa^	0.26 ± 0.05 ^ABa^
Long-chain fatty acids					
Palmitic (C16:0)	1	29.55 ± 0.61 ^ABa^	30.53 ± 0.24 ^Ab^	30.24 ± 0.94 ^Aa^	28.21 ± 0.54 ^Bb^
	21	30.68 ± 1.06 ^Ba^	33.63 ± 0.10 ^Aa^	29.59 ± 0.32 ^Ba^	30.51 ± 0.61 ^Ba^
Palmitoleic (C16:1) ω7	1	0.93 ± 0.01 ^Aa^	0.84 ± 0.06 ^Aa^	0.85 ± 0.06 ^Aa^	0.91 ± 0.00 ^Aa^
	21	1.08 ± 0.24 ^Aa^	0.71 ± 0.04 ^Ba^	0.76 ± 0.03 ^ABa^	0.93 ± 0.16 ^Aa^
Margaric (C17:0)	1	0.94 ± 0.04 ^Aa^	0.93 ± 0.15 ^Aa^	0.90 ± 0.01 ^Aa^	0.81 ± 0.13 ^Aa^
	21	0.88 ± 0.04 ^Aa^	0.81 ± 0.06 ^Aa^	0.81 ± 0.00 ^Aa^	0.92 ± 0.12 ^Aa^
Heptadecanoic (C17:1)	1	0.30 ± 0.04 ^Aa^	0.26 ± 0.02 ^Aa^	0.22 ± 0.05 ^Aa^	0.28 ± 0.02 ^Aa^
	21	0.23 ± 0.01 ^Aa^	0.26 ± 0.02 ^Aa^	0.22 ± 0.06 ^Aa^	0.25 ± 0.17 ^Aa^
Stearic (C18:0)	1	11.28 ± 0.07 ^Aa^	11.33 ± 0.74 ^Aa^	11.82 ± 0.51 ^Aa^	10.89 ± 0.04 ^Aa^
	21	10.62 ± 0.75 ^Aa^	10.99 ± 0.20 ^Aa^	11.87 ± 0.46 ^Aa^	11.19 ± 0.47 ^Aa^
Elaidic (C18:1 n9trans) ω9	1	2.79 ± 0.01 ^Aa^	2.85 ± 0.04 ^Aa^	2.45 ± 0.45 ^Aa^	2.56 ± 0.01 ^Aa^
	21	2.38 ± 0.52 ^Aa^	2.44 ± 0.17 ^Aa^	2.77 ± 0.02 ^Aa^	2.15 ± 0.07 ^Aa^
Oleic (C18:1 n9cis) ω9	1	17.64 ± 0.58 ^Aa^	17.37 ± 0.51 ^ABa^	17.35 ± 0.49 ^ABa^	16.18 ± 0.14 ^Ba^
	21	16.90 ± 1.59 ^ABa^	16.52 ± 0.50 ^Ba^	18.86 ± 0.31 ^Aa^	16.87 ± 0.21 ^ABa^
Linolelaidic (C18:2 n6trans) ω6	1	0.18 ± 0.01 ^Aa^	0.12 ± 0.02 ^Bb^	0.15 ± 0.02 ^ABa^	0.08 ± 0.02 ^Ba^
	21	0.19 ± 0.15 ^Ba^	0.50 ± 0.10 ^Aa^	0.27 ± 0.08 ^ABa^	0.15 ± 0.03 ^Ba^
Linoleic (C18:2 n6cis) ω6	1	2.91 ± 0.05 ^Aa^	2.89 ± 0.05 ^Aa^	2.94 ± 0.08 ^Aa^	2.81 ± 0.01 ^Aa^
	21	2.74 ± 0.38 ^ABa^	2.48 ± 0.05 ^Ba^	3.21 ± 0.04 ^Aa^	2.41 ± 0.34 ^Ba^
Arachidic (C20:0)	1	0.51 ± 0.04 ^ABa^	0.40 ± 0.16 ^Ba^	0.59 ± 0.01 ^ABa^	0.73 ± 0.16 ^Aa^
	21	0.40 ± 0.03 ^Aa^	0.20 ± 0.04^A Bb^	0.25 ± 0.09^A Bb^	0.29 ± 0.03 ^Ab^
Gamma-linolenic (C18:3 n6cis) ω6	1	0.17 ± 0.02 ^Aa^	0.17 ± 0.01 ^Aa^	0.12 ± 0.03 ^Ba^	0.17 ± 0.01 ^Aa^
	21	0.16 ± 0.01 ^Aa^	0.34 ± 0.23 ^Aa^	0.20 ± 0.05 ^Aa^	0.20 ± 0.05 ^Aa^
Gondoic (C20:1 n11cis) ω9	1	0.90 ± 0.01 ^Aa^	0.77 ± 0.06 ^ABa^	0.64 ± 0.15 ^Ba^	0.79 ± 0.03 ^ABa^
	21	0.67 ± 0.08^A Bb^	0.54 ± 0.12 ^Bb^	0.68 ± 0.07 ^ABa^	0.83 ± 0.04 ^Aa^
Alpha-linolenic (C18:3 n9cis) ω3	1	nd	nd	0.06 ± 0.01 ^Aa^	nd
	21	0.04 ± 0.01 ^Ba^	0.13 ± 0.02 ^Aa^	0.06 ± 0.02 ^Ba^	0.07 ± 0.01 ^Ba^
Heneicosylic (C21:0)	1	0.11 ± 0.04 ^Aa^	0.06 ± 0.01 ^ABa^	0.06 ± 0.01^A Bb^	0.05 ± 0.00 ^Bb^
	21	0.08 ± 0.00 ^Ba^	0.11 ± 0.04 ^Ba^	0.19 ± 0.12 ^Ba^	0.79 ± 0.04 ^Aa^
Behenic (C22:0)	1	0.94 ± 0.19 ^Ba^	1.03 ± 0.44 ^ABa^	1.06 ± 0.16 ^ABa^	1.66 ± 0.18 ^Aa^
	21	1.10 ± 0.02 ^Aa^	0.50 ± 0.32 ^Aa^	0.85 ± 0.10 ^Aa^	0.81 ± 0.62 ^Ab^
Erucic (C22:1n9) ω9	1	0.28 ± 0.02 ^Aa^	0.22 ± 0.02 ^Ba^	0.21 ± 0.02 ^Ba^	0.22 ± 0.01 ^Ba^
	21	0.18 ± 0.04^A Bb^	0.16 ± 0.02 ^ABa^	0.22 ± 0.03 ^Aa^	0.08 ± 0.06 ^Bb^
Eicosapentaenoic (C20:5)	1	2.32 ± 0.52 ^Ba^	1.97 ± 0.25 ^Ba^	2.67 ± 0.16 ^Ba^	4.67 ± 0.47 ^Aa^
	21	2.54 ± 0.23 ^Aa^	1.68 ± 0.06 ^Ba^	2.31 ± 0.24 ^Aa^	2.31 ± 0.03 ^Ab^
Docosahexaenoic (C22:6)	1	0.63 ± 0.18 ^Ba^	0.37 ± 0.16 ^Bb^	0.68 ± 0.04 ^Ba^	1.32 ± 0.16 ^Aa^
	21	0.74 ± 0.12 ^Ba^	1.11 ± 0.08 ^Aa^	0.72 ± 0.09 ^Ba^	0.51 ± 0.12 ^Bb^
SFA	1	70.25 ± 0.12 ^ABa^	71.68 ± 0.53 ^ABa^	72.64 ± 2.01 ^Aa^	69.64 ± 0.19 ^Ba^
	21	70.91 ± 2.15 ^Aa^	72.64 ± 2.01 ^Aa^	69.22 ± 0.01 ^Aa^	72.61 ± 0.46 ^Aa^
UFA	1	29.76 ± 0.12 ^ABa^	28.32 ± 0.53 ^ABa^	27.36 ± 2.01 ^Ba^	30.36 ± 0.19 ^Aa^
	21	29.10 ± 2.16 ^Aa^	27.36 ± 2.01 ^Aa^	30.79 ± 0.01 ^Aa^	27.39 ± 0.46 ^Aa^
MUFA	1	23.57 ± 0.55 ^Aa^	22.82 ± 0.58 ^Aa^	21.14 ± 1.77 ^Aa^	21.30 ± 0.42 ^Aa^
	21	22.71 ± 1.28 ^Aa^	21.14 ± 1.77 ^Aa^	24.04 ± 0.31 ^Aa^	21.75 ± 0.59 ^Aa^
PUFA	1	6.18 ± 0.68 ^Ba^	5.50 ± 0.05 ^Ba^	6.22 ± 0.23 ^Ba^	9.06 ± 0.61 ^Aa^
	21	6.39 ± 0.88 ^Aa^	6.22 ± 0.23 ^Aa^	6.75 ± 0.31 ^Aa^	5.64 ± 0.13 ^Ab^
AI	1	2.40 ± 0.04 ^ABa^	2.56 ± 0.01 ^Aa^	2.62 ± 0.18 ^Aa^	2.28 ± 0.04 ^Ba^
	21	2.60 ± 0.41 ^Aa^	2.76 ± 0.22 ^Aa^	2.28 ± 0.02 ^Aa^	2.69 ± 0.02 ^Aa^
TI	1	2.20 ± 0.23 ^Aa^	2.48 ± 0.03 ^Aa^	2.20 ± 0.08 ^Aa^	1.51 ± 0.10 ^Bb^
	21	2.17 ± 0.24 ^Ba^	2.48 ± 0.08 ^Aa^	2.13 ± 0.08 ^Ba^	2.35 ± 0.06 ^ABa^
DFA	1	40.04 ± 0.05 ^Aa^	39.65 ± 0.21 ^ABa^	39.18 ± 1.50 ^Ba^	41.25 ± 0.23 ^Aa^
	21	39.72 ± 2.91 ^Aa^	38.35 ± 2.20 ^Aa^	42.66 ± 0.46 ^Aa^	38.58 ± 0.01 ^Aa^
HSFA	1	42.53 ± 0.68 ^Aa^	43.60 ± 0.19 ^Aa^	42.78 ± 0.27 ^Aa^	41.02 ± 0.54 ^Ba^
	21	44.38 ± 2.63 ^ABa^	46.41 ± 0.26 ^Aa^	42.16 ± 0.43 ^Ba^	44.07 ± 0.91 ^ABa^

Results are expressed as average (*n* = 9) ± standard deviation. Abbreviations: SFA—saturated fatty acid; UFA—unsaturated fatty acid; MUFA—monounsaturated fatty acid; PUFA—polyunsaturated fatty acid; AI—atherogenicity index; TI—thrombosis index; DFA—desirable fatty acid; HSFA—hypercholesterolemic saturated fatty acid; nd—not detected. AI = (C12:0 + 4 C14:0 + C16:0)/[ΣMUFA + ΣPUFA(n − 6) and (n − 3)]; TI = (C14:0 + C16:0 + C18:0)/[0.5 × ΣMUFA + 0.5 × ΣPUFA(n − 6) + 3 × ΣPUFA(n − 3) + (n − 3)/(n − 6)]; DFA = MUFA + PUFA + C18:0; HSFA = C12:0 + C14:0 + C16:0. Formulations: CC—goat cream cheese without probiotic and xique-xique flour (control); PC—goat cream cheese with probiotic *Lactiplantibacillus plantarum* CNPC003; XC—goat cream cheese with xique-xique flour; PXC—goat cream cheese with probiotic *Lactiplantibacillus plantarum* CNPC003 and xique-xique flour. ^A,B^ Average ± standard deviation with different capital letters on same line differed based on Tukey’s test (*p* < 0.05) among cheese formulations. ^a,b^ Mean ± standard deviation with different lowercase letters in same column differed based on Student’s *t*-test (*p* < 0.05) during storage.

**Table 6:** Volatile compounds in goat cream cheese on days 1 and 21 of refrigerated storage.

Class	Compounds	IR Lit	IR	Storage/Days	CC	PC	XC	PXC
Aldehyde	Trans-2-decenal	1263	1263	1	<LOD	<LOD	<LOD	0.52 ± 0.03
				21	<LOD	<LOD	<LOD	<LOD
Acid	Acetic acid	610	<800	1	12.65 ± 0.84 ^Ba^	21.20 ± 0.60 ^Aa^	16.08 ± 0.35 ^Ba^	19.46 ± 3.29 ^ABb^
				21	22.73 ± 2.66 ^Ba^	33.65 ± 19.3 ^Ba^	20.57 ± 8.16 ^Ba^	78.36 ± 8.18 ^Aa^
	Isopentanoic acid	901	904	1	<LOD	<LOD	<LOD	<LOD
				21	<LOD	<LOD	<LOD	13.87 ± 0.09
	Ethylmethylacetic acid	898	913	1	<LOD	<LOD	<LOD	<LOD
				21	<LOD	<LOD	<LOD	12.92 ± 1.62
	Hexanoic acid	990	1000	1	<LOD	<LOD	1.93 ± 0.08 ^Ba^	3.83 ± 0.75 ^A^
				21	1.59 ± 0.38 ^Aa^	<LOD	1.23 ± 0.96 ^Aa^	<LOD
	Octanoic acid	1180	1185	1	4.64 ± 0.82 ^Ba^	6.47 ± 0.02 ^AB^	9.27 ± 2.42 ^Aa^	5.23 ± 1.13 ^Ba^
				21	8.16 ± 0.84 ^Aa^	<LOD	6.68 ± 1.37 ^Aa^	7.65 ± 0.99 ^Aa^
	Decanoic acid	1373	1373	1	6.72 ± 3.16 ^Aa^	4.73 ± 0.78 ^Aa^	5.73 ± 0,15 ^Aa^	4.26 ± 2.32 ^Aa^
				21	4.28 ± 1.09 ^Aa^	6.38 ± 1.98 ^Aa^	4.48 ± 3.23 ^Aa^	4.26 ± 2.13 ^Aa^
Alcohol	2-Ethylhexanol	1030	1033	1	0.90 ± 0.05 ^Aa^	0.63 ± 0.13 ^Ba^	1.02 ± 0.07 ^Aa^	0.46 ± 0.07 ^Bb^
				21	1.12 ± 0.01 ^Aa^	0.71 ± 0.05 ^Ba^	1.07 ± 0.10 ^Aa^	0.84 ± 0.11 ^Ba^
	1-Octanol	1071	1075	1	1.30 ± 0.08 ^Ba^	1.96 ± 0.16 ^A^	1.40 ± 0.02 ^Ba^	1.73 ± 0.13 ^Ab^
				21	1.39 ± 0.18 ^Ba^	<LOD	1.06 ± 0.05 ^Bb^	2.40 ± 0.34 ^Aa^
	1-Nonanol	1173	1174	1	<LOD	0.49 ± 0.01 ^Aa^	0.52 ± 0.15 ^A^	0.55 ± 0.17 ^Aa^
				21	<LOD	0.58 ± 0.04 ^Aa^	<LOD	0.57 ± 0.04 ^Aa^
	1-Decanol	1273	1274	1	2.76 ± 1.60 ^A^	<LOD	<LOD	0.38 ± 0.02 ^Ba^
				21	<LOD	<LOD	<LOD	0.49 ± 0.05 ^a^
Ketone	Acetoin	713	<800	1	0.83 ± 0.14 ^Ba^	3.00 ± 0.73 ^Aa^	0.82 ± 0.06 ^Ba^	2.15 ± 0.01 ^Aa^
				21	2.37 ± 1.71 ^Aa^	5.77 ± 4.13 ^Aa^	2.22 ± 1.34 ^Aa^	6.31 ± 2.33 ^Aa^
	2-Heptanone	891	891	1	3.00 ± 0.16 ^Aa^	1.69 ± 0.14 ^B^	2.85 ± 0.16 ^Aa^	1.42 ± 0.18 ^Ba^
				21	3.94 ± 1.40 ^Aa^	<LOD	3.02 ± 1.38 ^Aa^	1.90 ± 1.12 ^Aa^
Terpene	α-Copaene	1376	1379	1	<LOD	<LOD	0,34 ± 0.07 ^a^	<LOD
				21	<LOD	<LOD	0,75 ± 0.09 ^Aa^	<LOD
	β-Caryophyllene	1419	1423	1	<LOD	0.60 ± 0.08 ^Aa^	1.37 ± 0.83 ^Ab^	1.67 ± 0.58 ^Aa^
				21	<LOD	1.51 ± 0.82 ^Ba^	3.06 ± 0.03 ^Aa^	2.37 ± 0.34^A Ba^
	(+)-δ-Cadinene	1524	1527	1	<LOD	<LOD	0.25 ± 0.09 ^a^	<LOD
				21	<LOD	<LOD	0.25 ± 0.01 ^a^	<LOD
Hidrocarboneto	1-Decyne	-	1027	1	2.78 ± 0.14 ^Aa^	5.38 ± 4.21 ^Aa^	2.42 ± 1.70 ^Aa^	1.04 ± 0.24 ^Ab^
				21	5.55 ± 0.15 ^Aa^	2.51 ± 0.83 ^Aa^	7.12 ± 4.44 ^Aa^	10.29 ± 8.29 ^Aa^

Results are expressed as average (*n* = 9) ± standard deviation. Average values of volatile compounds identified in goat cream cheese samples over 21 days of storage. Values are expressed in peak counts. IR Lit: literature retention index. IR: calculated retention index. Abbreviation: <LOD—Below limit of detection (LOD). Formulations: CC—goat cream cheese without probiotic (control); PC—goat cream cheese with probiotic *Lactiplantibacillus plantarum* CNPC003; XC—goat cream cheese with xique-xique flour; PXC—goat cream cheese with probiotic *Lactiplantibacillus plantarum* CNPC003 and xique-xique flour. ^A,B^ Average ± standard deviation with different capital letters on same line differed based on Tukey’s test or Student’s *t*-test (*p* < 0.05) among cheese formulations. ^a,b^ Average ± standard deviation with different lowercase letters in same column differed based on Student’s *t*-test (*p* < 0.05) during storage.

**Table 7:** Just about right (JAR) results for goat cream cheese formulations.

Attributes	Formulations			
	CC	PC	XC	PXC
Color	3.26 ± 0.56 ^a^	3.00 ± 0.33 ^ab^	2.84 ± 0.69 ^b^	2.58 ± 0.61 ^b^
Goat aroma	3.42 ± 0.61 ^a^	3.53 ± 0.91 ^a^	3.32 ± 0.82 ^a^	3.21 ± 0.63 ^a^
Herbaceous aroma	NA	NA	3.53 ± 0.51 ^a^	3.05 ± 0.71 ^a^
Consistency	3.05 ± 0.97 ^a^	2.84 ± 0.83 ^a^	2.95 ± 0.62 ^a^	3.00 ± 0.47 ^a^
Texture	3.32 ± 0.67 ^a^	2.89 ± 0.46 ^ab^	3.11 ± 0.57 ^a^	2.79 ± 0.57 ^b^
Salt	2.95 ± 0.71 ^a^	3.16 ± 0.77 ^a^	3.00 ± 0.68 ^a^	2.95 ± 0.78 ^a^
Acidity	3.32 ± 0.67 ^b^	4.00 ± 0.58 ^a^	3.89 ± 0.99 ^a^	3.95 ± 0.62 ^a^
Herbaceous flavor	NA	NA	3.74 ± 0.99 ^a^	3.58 ± 0.90 ^a^

Results are expressed as average (*n* = 102) ± standard deviation. Abbreviation: NA—not applicable. Formulations: CC—goat cream cheese without probiotic (control); PC—goat cream cheese with probiotic *Lactiplantibacillus plantarum* CNPC003; XC—goat cream cheese with xique-xique flour; PXC—goat cream cheese with probiotic *Lactiplantibacillus plantarum* CNPC003 and xique-xique flour. ^a,b^ Average ± standard deviation with different lowercase letters on same line differed by Tukey’s test or Student’s *t*-test (*p* < 0.05) among cheese formulations (*n* = 12). JAR on 5-point scale (1 = extremely less than ideal; 5 = extremely greater than ideal).

## Data Availability

The data can be made available upon request.
